# Meta-analysis of genomic characteristics for antiviral influenza defective interfering particle prioritization

**DOI:** 10.1093/nargab/lqaf031

**Published:** 2025-04-04

**Authors:** Jens J G Lohmann, Mia Le, Fadi G Alnaji, Olga Zolotareva, Jan Baumbach, Tanja Laske

**Affiliations:** Institute for Computational Systems Biology, University of Hamburg, 22761 Hamburg, Germany; Institute for Computational Systems Biology, University of Hamburg, 22761 Hamburg, Germany; Department of Virology, Bernhard Nocht Institute for Tropical Medicine, 20359 Hamburg, Germany; A*STAR Infectious Diseases Labs (A*STAR ID Labs), Agency for Science, Technology and Research (A*STAR), 8A Biomedical Grove, Immunos #05-13, 138648 Singapore, Singapore; Institute for Computational Systems Biology, University of Hamburg, 22761 Hamburg, Germany; Institute for Computational Systems Biology, University of Hamburg, 22761 Hamburg, Germany; Computational Biomedicine Lab, Department of Mathematics and Computer Science, University of Southern Denmark, 5230 Odense, Denmark; Institute for Computational Systems Biology, University of Hamburg, 22761 Hamburg, Germany; Viral Systems Modeling, Leibniz Institute of Virology, 20251 Hamburg, Germany

## Abstract

Defective interfering particles (DIPs) are viral deletion mutants that hamper virus replication and are, thus, potent novel antiviral agents. To evaluate possible antiviral treatments, we first need to get a deeper understanding of DIP characteristics. Thus, we performed a meta-analysis of 20 already published sequencing datasets of influenza A and B viruses (IAV and IBV) from *in vivo* and *in vitro* experiments. We analyzed each dataset for characteristics, such as deletion-containing viral genome (DelVG) length distributions, direct repeats, and nucleotide enrichment at the deletion site. Our analysis suggests differences in the length of the 3′- and 5′-end retained in IAV and IBV viral sequences upon deletion. Moreover, *in vitro* DelVGs tend to be shorter than those *in vivo*, which is a novel finding with potential implications for future DIP treatment design. Additionally, our analysis demonstrates the presence of DelVGs with longer than expected sequences, possibly related to an alternative mechanism of DelVG formation. Finally, a joint ranking of DelVGs originating from 7 A/Puerto Rico/8/1934 datasets revealed 11 highly abundant, yet unnoticed, candidates. Together, our study highlights the importance of meta-analyses to uncover yet unknown DelVG characteristics and to pre-select candidates for antiviral treatment design.

## Graphical Abstract

**Graphical Abstract:**
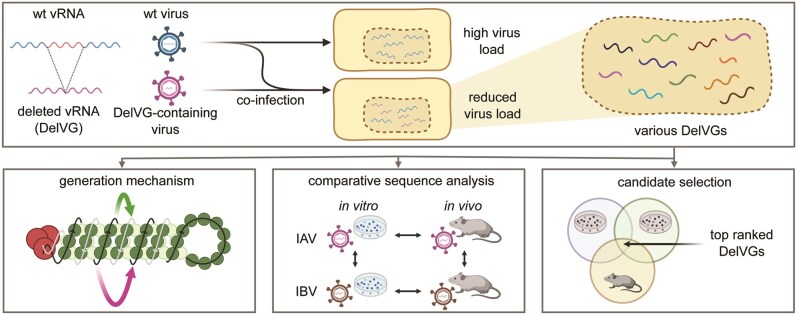

## Introduction

Defective interfering particles (DIPs) are replication-incompetent virus particles that arise naturally in virus populations, due to errors in replication. DIPs have been described in most known virus families and are most commonly present as deleted, sub-genomic viral sequences (reviewed by [1–6]). In this study, we focus on influenza viruses, which are negative-sense RNA viruses with a segmented genome. An influenza virus DIP carries at least one truncated genome segment, which replaces its cognate full-length segment during genome packaging [7–9]. Furthermore, two or more segments can be replaced by defective segments in one particle (reviewed by Brooke [10]). Because of the defective segment, DIPs cannot produce all viral proteins needed for propagation. Thus, they are dependent on the co-infection by its standard virus to complement the missing viral proteins and complete the replication cycle. During co-infection, DIPs interfere with standard virus replication by competitive inhibition, reducing the number of standard virus particles. Since it is unknown if all deletions result in an interfering RNA, we refer to them as deletion-containing viral genomes (DelVGs) as proposed by Alnaji and Brooke [11]. Different types of DelVGs, such as single deletion, double deletion, and mosaic DelVGs are described for influenza viruses [12]. The single deletion DelVG, which is considered in this study, contains a large internal deletion of the genomic segment, retaining only the 3′- and 5′-ends of the RNA sequence [7, 12, 13]. The occurrence of the single deletion DelVG is explained by the following mechanism: the polymerase stops copying the RNA at one position and continues at a position further downstream of the RNA sequence template [12, 14]. The most common model to explain this mechanism is referred to as polymerase template switch, while, other models are being discussed in the literature currently [11, 15].

Due to their ability to interfere with virus replication and reduce the number of infectious virus particles, the application of DIPs as a novel antiviral agent has been investigated since the 1980s [16]. In particular, animal studies [17–20] have demonstrated their potential antiviral effect, making them greatly interesting for medical application [21–23]. One possibility would be to produce a DelVG with high antiviral potential and treat an infected patient. The DelVG will interfere with the virus and eventually support viral clearance by reducing the infectious virus load in the patient. To enable such antiviral treatments, a first step is to assess and select potent antiviral DelVG candidates. A potent antiviral DelVG candidate is one that outcompetes others and enriches under high selective pressure, e.g. during repeated virus passaging. For instance, Pelz *et al.* [24] have successfully shown that enriched candidates reduce the virus titer upon infection significantly more than other candidates.

By now, it is unknown which molecular characteristics lead to the distinct virus titer reduction, which could be elucidated by a systematic analysis of DelVG sequence features. One important feature is the DelVG’s segment origin. For influenza viruses, typically, DelVGs originate from one of the three polymerase-encoding segments (PB2, PB1, and PA), which are the longest of the eight coding RNA segments [17, 18]. Based on this observation, it has been proposed that the number of DelVGs per segment could be proportional to the segment length [7]. Other factors with possible impact on the generation and/or enrichment of DelVGs are direct (sequence) repeats [25–28] and nucleotides enriched around the deletion site [27, 29]. Furthermore, it is hypothesized that other factors like motifs and other regulatory effects could also play a role in the formation of the deletion site [7, 29].

Due to their clinical potential, DelVGs have acquired significant global attention, leading to the publication of numerous studies examining their characteristics. While these studies have substantially expanded our understanding of DelVGs, they were conducted under varying conditions, including different virus strains, host systems, and infection times, resulting in some inconsistencies in the reported data. To enable more detailed and unbiased comparisons across these studies, we performed a systematic meta-analysis on 20 published datasets. The datasets were collected by a database search and the single deletion DelVGs were identified using a pipeline by Alnaji *et al.* [28]. Other types of DelVGs, such as double deletion, mosaic, or copy-back were not in the scope of this meta-analysis. By incorporating datasets generated using different influenza virus strains, experimental conditions (*in vitro* or *in vivo*), and cell lines, we aim to comprehensively characterize and compare DelVGs. The found characteristics were validated by comparing them with synthetic datasets, generated by randomly sampling DelVGs including knowledge-based restrictions. Furthermore, we identify several DelVG candidates, which have a consistently high prevalence in independent infection experiments. Since a high abundance of DelVGs has been previously associated with a high antiviral activity [24], such DelVG candidates could be promising in medical applications [24]. Top DelVG candidates were identified by intersecting the DelVGs of seven datasets of different sources, all containing the influenza virus A/Puerto Rico/8/1934 strain. The approach is validated by demonstrating that the number of occurrences of a DelVG in multiple datasets is higher than expected based on a randomly sampled synthetic dataset.

## Materials and methods

### Identification of relevant publications

To select relevant publications and datasets for the meta-analysis, three queries were performed according to the PRISMA guidelines [30]. We searched PubMed to identify relevant publications in the field [query: (“influenza”[Title] AND “defective”[Title]), date: 8.11.2023]. Additionally, we screened the BioProject database with two independent queries to find sequencing data [query 1: (“influenza b virus”), query 2: (“Puerto Rico 8”), both on 22.11.2023]. In total 246 entries were identified. Subsequently, we excluded duplicates, reviews, and publications without sufficient data. More detail about the number of records and exclusion criteria can be found in the PRISMA flow chart [30] (Supplementary Fig. S1). Overall, 16 relevant studies were identified [24, 26–29, 31–41]. The studies by Alnaji *et al.* [28], Berry *et al.* [33], and Valesano *et al.* [34] contained sequencing data of multiple influenza virus strains. The DelVGs of each strain are considered as an individual dataset, resulting in 22 datasets. The datasets were named by the corresponding author, combined with the publishing year. If one study contained different strains, a suffix for the strain was added to further distinguish the datasets. For the same publication, sequencing data from different time points or replicates were pooled and considered as one dataset. An overview of the datasets, including the accession numbers is given in Supplementary Table S1.

### DelVG identification and filtering

The DelVGs in the considered datasets were identified using a pipeline by Alnaji *et al.* [28]. The default parameter values of the pipeline were used. To run the pipeline it is necessary to provide the reference sequence of an influenza virus strain to which the reads are mapped. The strain given in the corresponding publication was considered for this. If no strain was provided, the metadata of the BioProject entry was used to identify a matching strain. The datasets and their corresponding reference genomes are listed in Supplementary Table S1 and S2. For Mendes2021 [38], we used reverse complements of the sequences provided by the authors (Supplementary Fig. S2).

To reduce the number of false-positive DelVGs, we apply a read support cutoff (RSC) to the identified DelVGs [28]. The RSC is a threshold value and controls the number of times a single DelVG sequence needs to occur in one dataset to be considered as relevant and thus be included in the analysis. In literature, the RSC is dataset-specific and varies between 5 [29] and 30 [27, 28]. To estimate an appropriate RSC for our analysis and allow a reasonable comparison of the datasets, we performed a systematic estimation of the RSC. For this estimation, we compared the original datasets that were accessible to us with the datasets processed by the DelVG identification pipeline [28] used in this meta-analysis (Supplementary Fig. S3 and Supplementary Table S3). The fraction of DelVGs that are found in both datasets was estimated for an increasing RSC until an identity of 75% was reached (Supplementary Fig. S3). Over all tested datasets, the mean RSC was 11.0 (Supplementary Table S3). Therefore, an RSC of 15 was applied on all processed datasets. Furthermore, we set a threshold of 40 distinct DelVGs as exclusion criteria to obtain adequate sample sizes for statistical analyses. Since >40 DelVGs were identified in the majority of datasets, finally, 20 datasets were considered in the analysis. Only the two datasets VdHoecke2015 [32] (eight DelVGs) and Kupke2020 [41] (five DelVGs) were excluded, because they contain too few distinct DelVGs.

### Random sampling approach to generate synthetic datasets

To estimate statistical significance of the findings, we compared results obtained from real-world next-generation sequencing (NGS) datasets to random synthetic datasets. The motivation to utilize synthetic data is to compare the observed data distributions against distributions of DelVGs that can theoretically emerge in a given experiment. Hence, we can investigate whether features of DelVG sequences were due to chance, i.e. random deletions (synthetic data), or represent significant findings with potential biological implications. Note that we consider only those theoretically possible virus strain-specific DelVGs relevant for a given experiment. Thus, the synthetic datasets include prior knowledge about the deletion positions and identifiability of direct repeats. These synthetic datasets were created for each dataset individually by sampling random deletion sites inside of hotspot areas [28] at the start and end of the respective reference sequence (Equations 1 and 2). The hotspot areas (“start” and “end”) were defined individually for each dataset by accounting for an upper and a lower bound based on the corresponding experimental results. The lower bound of the start position was set to the maximum of 50 nt and the mean of the start positions (*μ*_s_) − 200 nt (Equation 1). The upper bound was set to *μ*_s_ + 200 nt.


(1) \begin{eqnarray*}
{\rm start} = \left[ {{\rm max}\left( {50,{{\mu }_{\rm s}} - 200} \right),{{\mu }_{\rm s}} + 200} \right].
\end{eqnarray*}

The lower bound of the end positions was set to the mean of the end positions (*μ*_e_) − 200 nt and the upper bound was set to the minimum of *μ_e_* + 200 nt and the length of the sequence (*l*_seq_) − 50 nt (Equation 2). Sampling from these defined areas provides DelVGs that start and end at the termini of the sequence. Likewise, DelVGs that originate from the center of the sequence are excluded.


(2) \begin{eqnarray*}
{\rm end} = \left[ {{{\mu }_{\rm e}} - 200,{\rm min}\left( {{{\mu }_{\rm e}} + 200,{{l}_{{\rm seq}}} - 50} \right)} \right].
\end{eqnarray*}

All possible combinations of the defined start and end positions provide a set of theoretically possible DelVGs. Furthermore, we filtered out non-observable DelVGs based on the impact of so-called direct repeats [27]. A direct repeat is a repeated sequence at the start and end of the deletion sites, usually 1–5-nt long. The repeated sequences make it impossible to determine the exact positions of the deletion site which led to the observed DelVG. To handle this uncertainty, the used pipeline [28] shifts the deletion site either to the right or left side of the repeated sequence. To account for this shift in the synthetic datasets, we replaced all DelVGs that are not identifiable by the pipeline with the DelVG that is identified after applying the shift. After excluding these candidates for each of the eight influenza virus genome segments, 35 000 of the artificial DelVGs were randomly sampled with replacement for each segment. This number was estimated by a convergence analysis. Datasets of increasing size were sampled in steps of 1000. As convergence criterion, we used the relative occurrence of adenine at the position before the start of the deletion site. It was estimated at which dataset size five successive datasets had a standard deviation of less than 0.002 for the relative occurrence. This criterion was met latest at a dataset size of 34 000 (data not shown) for all segments in the datasets. Therefore, the overall number of data points per segment was set to 35 000.

### Statistical analyses

For the evaluation of the results different statistical tests were performed using the Python library SciPy (version 1.13.1).

#### Cliff’s delta

Cliff’s delta is a *post hoc* test to study the effect sizes after applying Wilcox–Mann–Whitney *U*-test. It compares the number of times the value of group one is higher than samples from group two. We are using a form of it that includes the *U* statistics from the Wilcox–Mann–Whitney test (Equation 3). The size of the two groups are given by *n*_1_ and *n*_2_:


(3) \begin{eqnarray*}
d = \frac{{2*{{U}_1}}}{{{{n}_1}*{{n}_2}}} - 1.
\end{eqnarray*}

Cliff’s delta ranges from −1 to 1. If no group dominates the other group, Cliff’s delta is 0. A positive value demonstrates that the first group dominates the second group, while a negative value indicates the opposite. Dominance refers here to the number of values from one group that is greater than or less than values from another group. The magnitude of Cliff’s delta corresponds to the strength of the observed effect. Meissel and Yao [42] have outlined thresholds for the effect size: negligible (<0.15), small (0.15–0.32), medium (0.33–0.46), and large (>0.46).

#### Scheirer–Ray–Hare test

The Scheirer–Ray–Hare test investigates if a measure is affected by two factors [43]. It builds upon the Kruskal–Wallis test and is therefore a nonparametric alternative to the two factor ANOVA. The test is used to investigate if two given factors have individually no influence on the measurement outcome. It tests the null hypothesis that the two factors have no interaction. Multiple testing correction was applied where applicable, which is detailed in the corresponding sections.

The Scheirer–Ray–Hare test is a rank-based test and therefore the ranks (*r*) of the measurement are assigned in a first step. Based on these ranks, the mean of squares (*MS*) is calculated (Equation 4). For each factor, different groups (*g*_1_ and *g*_2_) exist and based on these the squared deviation is calculated and multiplied by the number of observations of the group (*n_i_*) to get the sum of squares (*f1*_SS_ and *f2*_SS_) (Equations 5 and 6). Additionally, the within sum of squares (*w*_SS_) (Equation 7) and the total sum of squares are calculated (*t*_SS_) (Equation 8). With these, the interaction sum of squares (*i*_SS_) can be calculated, which is the *t*_SS_ minus the *w*_SS_ and the two factors (*f1*_SS_ and *f2*_SS_) (Equation 9). The H statistic for the two factors and the interaction can then be calculated by dividing each *SS* (e.g. *f1*_SS_) by the *MS* (Equations 10–12).


(4) \begin{eqnarray*}
MS = \frac{1}{n}\mathop \sum \limits_{i = 1}^n {{\left( {{{r}_i} - \bar{r}} \right)}^2},
\end{eqnarray*}

(5) \begin{eqnarray*}
f{{1}_{{\rm SS}}} = \mathop \sum \limits_{i = 1}^{g1} {{n}_i}*{{\left( {\overline {{{r}_i}} - \bar{r}} \right)}^2},
\end{eqnarray*}

(6) \begin{eqnarray*}
f{{2}_{{\rm SS}}} = \mathop \sum \limits_{j = 1}^{g2} {{n}_j}*{{\left( {\overline {{{r}_j}} - \bar{r}} \right)}^2},
\end{eqnarray*}

(7) \begin{eqnarray*}
{{w}_{{\rm SS}}} = \mathop \sum \limits_{i = 1}^{g1} \mathop \sum \limits_{j = 1}^{g2} {{\left( {{{r}_{ij}} - \overline {{{r}_{ij}}} } \right)}^2}*\left( {{\rm min}\left( {\left| {{{r}_{ij}}} \right|} \right) - 1} \right),
\end{eqnarray*}

(8) \begin{eqnarray*}
{{t}_{{\rm SS}}} = MS*{{n}_{{\rm obs}}},
\end{eqnarray*}

(9) \begin{eqnarray*}
{{i}_{{\rm SS}}} = {{t}_{{\rm SS}}} - {{w}_{{\rm SS}}} - f{{1}_{{\rm SS}}} - f{{2}_{{\rm SS}}},
\end{eqnarray*}

(10) \begin{eqnarray*}
{{H}_{f1}} = \frac{{f{{1}_{{\rm SS}}}}}{{MS}},
\end{eqnarray*}

(11) \begin{eqnarray*}
{{H}_{f2}} = \frac{{f{{2}_{{\rm SS}}}}}{{MS}},
\end{eqnarray*}

(12) \begin{eqnarray*}
{{H}_i} = \frac{{{{i}_{{\rm SS}}}}}{{MS}}.
\end{eqnarray*}

### DelVG distribution across the influenza virus genome segments

To estimate the distribution of the DelVGs across the eight segments, the number of DelVGs per segment was counted. From these counts, the percentage of DelVGs per segment was calculated. This was performed for each dataset independently. Expected data were generated using the assumption that the number of DelVGs per segment is directly correlated to the segment length. This means that the longer the parental segment is, the higher the number of DelVGs from this segment is. Thus, for each strain, the RNA sequence length of each segment was divided by the total length of the genome (Supplementary Fig. S4). To test if the observed and expected distributions are independent, a chi-squared test was employed on the DelVG counts. In the test, the segments are representing the different categories. For datasets with significant *P*-values (α = 0.05), we calculated and reported Cramer’s V [44] as effect size.

### Deletion-induced shift of the reading frame

A deletion retains the reading frame if the length of the deletion is divisible by 3, as the length of the codon is 3. To estimate the proportion of DelVGs that retain the reading frame, the length of the deletion site was calculated for each DelVG. The modulo operator was used on each calculated length to determine the remainder. A remainder of 0 is considered as “in-frame” deletion, a remainder of 1 as “shift + 1”, and a remainder of 2 as ”shift − 1”. A chi-square test was then employed to compare observed and expected frequencies of the frame shifts. In the expected scenario it was assumed that each of the options is equally observed. This leads to a probability of ⅓ for each of the options. In the chi-square test the three shift options are representing the different categories. Cramer’s V [44] was additionally calculated as effect size for datasets with significant *P*-values (α = 0.05).

### Direct repeats

Direct repeats are defined by the number of nucleotides that are identical between the sequence before the start and end of the deletion site (see "Results" section for visualization, Fig. 4A). For each DelVG, the number of identical nucleotides at these positions was calculated. Direct repeats >4 were combined into one category. This leads to six distinct categories for the length of the direct repeats (0, 1, 2, 3, 4, and >4). To verify the observed data, synthetic datasets were generated using the random sampling approach (see the "Random sampling approach to generate synthetic datasets" section). A chi-squared test, followed by Cramer’s V [44] for significant values, was used to estimate if the distributions of the observed and synthetic data are the same. To visualize the results, for each of the six categories, the difference of the fractions between the observed and synthetic data was calculated.

### Nucleotide enrichment

For each of the datasets, the enrichment of specific nucleotides around the deletion site was assessed. For that, a 10-nt window around the start and the end of the deletion site was defined (see "Results" section for visualization, Fig. 5A). It includes the 5 nt that remain in the DelVG sequence and 5 nt of the deleted sequence. For each DelVG, the nucleotides at those positions were identified and the nucleotide distribution of the dataset was estimated. The distributions were validated by comparing them to randomly sampled synthetic data (see the "Random sampling approach to generate synthetic datasets" section). At each position, statistical significance of the nucleotide abundance differences between the two groups (original data and synthetic data) was estimated using the Kruskal–Wallis test and the effect size η^2^ [45] was determined for the significant positions (*P* < 0.05). For visualization, the fraction of nucleotides at each position was subtracted from the fraction of nucleotides that occurred at the same position in the synthetic data.

Additionally, the nucleotide pair of position 4 and 5 at the start and end of the deletion site was inspected further. For each DelVG, this nucleotide pair was constructed at the start and end position. The most frequent nucleotide pair was determined for each dataset, and its proportional occurrence in the dataset was calculated. To validate the results, the same procedure was applied on synthetic data that were generated using the random sampling approach described earlier (see the "Random sampling approach to generate synthetic datasets" section).

### Relevant DelVG identification

To identify potentially interesting DelVGs relevant for fundamental research, e.g. for DelVG formation and selection, or for clinical application, we focused on DelVGs that occur repeatedly in independent experiments and in the highest frequency. We based our approach on findings by Pelz *et al.* who showed that DelVGs that enrich over time in virus populations can reduce virus titers to a larger extent compared with other DelVGs [24]. To identify relevant DelVGs, the most frequent DelVGs of multiple datasets from the same strain were identified. For this, the seven NGS datasets from A/Puerto Rico/8/1934 infections were considered, which comprise the majority of datasets in our meta-analysis. Two of the seven datasets (Kupke2020 [41] and VdHoecke2015 [32]) were not considered in the full analysis as they contain <40 DelVGs, after applying an RSC of 15. It is still sufficient to include them in this analysis, because less relevant DelVGs will be filtered out during the analysis.

First, we calculated the fraction of intersecting DelVGs for each dataset pair. To validate these fractions, they were also calculated for randomly generated synthetic datasets (see the "Random sampling approach to generate synthetic datasets" section). The size of the experimental dataset was taken into account to generate equally large synthetic datasets. In a next step, DelVGs that occur in at least half of the datasets, which are four in this case, were selected. For each dataset, the percentile of the NGS read count for each DelVG was calculated. Based on these percentiles, two scores were defined for ranking the remaining DelVGs. The first is the sum of all percentiles. Using the sum as score favors DelVGs that occur in many datasets with a high NGS count. The second score is the mean of the percentiles, but omitting datasets that do not include the DelVG. This approach allows the detection of DelVGs that have a high NGS count. DelVGs that occur in the top *n* DelVGs of each scoring method were considered as final DelVG selection. The finally selected DelVGs were investigated in more detail by reviewing the label they were assigned to in Pelz *et al.* [24] and calculating other features used in the meta-analysis, such as direct repeat length.

### R shiny app

The analyses in this meta-analysis are made available in the R shiny DIP-DSA (Deletion Site Analyzer) application. It provides analyses for all DelVG features assessed in the present meta-analysis. It offers the possibility to inspect single datasets and also to compare multiple datasets with each other. The source code is available at Zenodo (https://doi.org/10.5281/zenodo.12157628) or GitHub (https://github.com/viraidip/DIP-DSA). Additionally, the application can be accessed online (https://apps.cosy.bio/dipdsa/).

## Results

### DelVGs are unequally distributed over the eight RNA segments for all datasets

**Figure 1.**
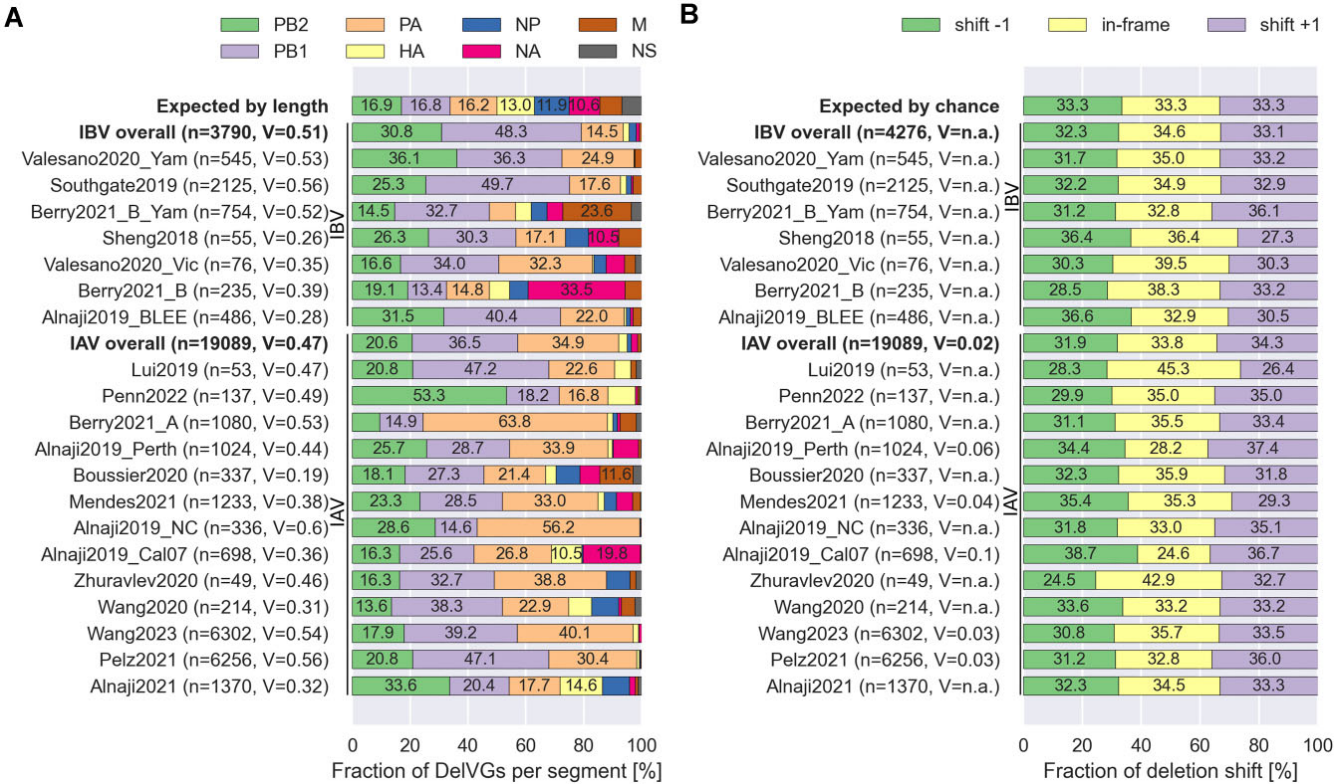
Origin of DelVGs and reading frame shifts. (**A**) Distribution of the segments across the DelVGs. It was assessed if the observed distribution is similar to a distribution that would be expected if the DelVGs occur solely dependent on the RNA sequence length (see the “DelVG distribution across the influenza virus genome segments” section). The top bar shows the mean of the expected values calculated over all datasets for reference. Statistical significance was assessed using a one-way chi-squared test and Cramer’s V [44] as effect size. (**B**) Distribution of the reading frame shift introduced by the deletion. For each dataset, a chi-squared test with Cramer’s V [44] as effect size was performed, compared with an expected distribution where each of the shifts occurs equally often. (Cramer’s V effect sizes [46]: V < 0.1 = negligible; 0.1 < V < 0.2 = weak; 0.2 < V < 0.4 = moderate; 0.4 < V < 0.6 = relatively strong; 0.6 < V < 0.8 = strong; 0.8 < V < 1.0 = very strong; n.a.: no significant *P*-value).

For influenza A and B virus (IAV and IBV) strains, DelVGs from the three polymerase-encoding segments are the most abundant (Fig. 1A). Although HA was observed to form high-abundance DelVGs comparable to the polymerase at an early stage (3–6 h post infection) in one study [29], further research is required to understand the dynamics of formation across temporal scales. In the 20 datasets the polymerase DelVGs combined make up a fraction ranging from 47.3% in Alnaji2019_BLEE [28] to 99.4% in Alnaji2019_NC [28]. But the distribution of the three polymerase-encoding segments is different between the datasets. For instance, PA-derived DelVGs are, with 63.8%, the most predominant DelVG type in Berry2021_A [33], while PB1-DelVGs represent the most abundant DelVGs in Lui2019 [26] and Pelz2021 [24]. In addition, PB1-derived DelVGs are also the most abundant in the IBV datasets Valesano2020_Yam and Berry2021_B_Yam. Notably, Alnaji2019_BLEE [28] is the only dataset where the segment with the most abundant DelVGs is not one of the polymerase-encoding segments, with NA-derived DelVGs contributing 33.5% of all candidates. Since other IBV datasets do not show this pattern, the high abundance of NA delVGs might be specific to the B/Lee/1940 strain. To rule-out that this observation is not experiment-specific, more B/Lee/1940 datasets would be required, however, they are currently not available. In the Alnaji2019_Cal07 [28] dataset, DelVGs from NA (19.8%) surpass those from PB2 (16.3%). Likewise, in the Sheng2018 [36] dataset, DelVGs from the M segment (23.6%) occur more frequently than those from PB2 (14.5%) and PA (9.1%). Overall, it is noteworthy that all datasets deviate significantly from the expected distribution of DelVGs based solely on RNA sequence length (Supplementary Fig. S4).

### In-frame deletions are not favored in influenza virus DelVGs

To assess whether the deletion sites correlate with the resulting polypeptide sequence, the prevalence of in-frame deletions was examined (see the "Deletion-induced shift of the reading frame" section). In five of the analyzed datasets, a significant difference of deletions was observed, but only with neglectable effect sizes (Cramer’s V ≤ 0.1) [46] (Fig. 1B). The three potential shift options (shift −1, in-frame, and shift +1) are equally distributed, with an approximate occurrence of one-third for each of the options. This suggests that maintaining the reading frame is not a critical factor for influenza virus DelVGs. However, it does not rule out the role of the truncated proteins produced by DelVGs [47, 48]. It is important to note that this observation does not extend to DelVGs in other viruses. For instance, Rezelj *et al.* identified a hotspot region in Flaviviruses where most DelVGs preserved the reading frame [49]. In contrast to influenza viruses, Flaviviruses possess a non-segmented genome, which could provide an explanation for this divergent observation.

### DelVG sequence length depends on the host system

**Figure 2.**
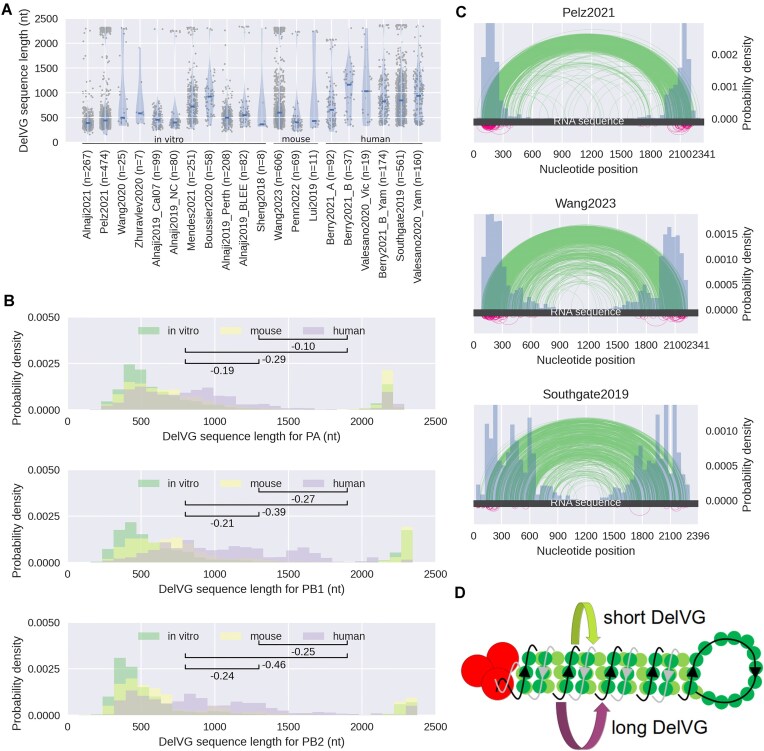
Length of the polymerase-encoding genes. (**A**) DelVG sequence lengths of PB2-derived DelVGs as a violin plot. The horizontal bar represents the median of the distribution. (**B**) Comparison of the DelVG sequence length between *in vitro*, *in vivo* mouse, and *in vivo* human groups (upper panel: PA, middle panel: PB1, and lower panel: PB2). The *y*-axis shows the probability density. The probability density is the raw counts divided by the sum of all counts multiplied with the bin width. By this, the area under the bins integrates to 1. Cliff’s delta was calculated for all significant *P*-values of the Wilcoxon–Mann–Whitney *U*-test (Supplementary Table S5) to estimate the effect of the groups on the DelVG lengths. Absolute effect sizes are considered negligible (<0.15), small (0.15–0.32), medium (0.33–0.46), or large (>0.46). (**C**) Mapping of the deletion start and end point positions on the reference genome of segment PB2. Distribution of the deletion site start and end positions is given in a histogram in blue. Long DelVGs with a sequence length of at least 85% of the full sequence are plotted below the RNA sequence bar (pink). The other DelVGs are plotted above the RNA sequence bar (green). (**D**) Hypothesis for the generation of the long DelVGs. The upper arrow (green) indicates the most common template switch that leads to very short DelVGs. The lower arrow (pink) indicates the template switch of the polymerase that creates the long DelVGs, potentially within the major groove of the vRNP.

In the literature, it is described that deletions occur more often at the start and end and rarely in the center of the segment’s RNA sequence [28]. This leads to the enrichment of rather short DelVG candidates. Previously, the average length of DelVGs originating from one of the polymerase-encoding segments was reported in a range of 400–500 nt [18, 22, 25]. In our analysis, the median DelVG length ranged from 386 to 1069 nucleotides in Sheng2018 [36] and Valesano2020_Yam [34], respectively (Fig. 2A). Interestingly, we found that DelVGs from *in vivo* experiments are, on average, 164 nt longer than from the *in vitro* experiments. Hence, we conducted further analysis with respect to the host system, accounting separately for the pooled datasets from cell culture (*in vitro*), living mice (mouse), or patients (human) (Supplementary Table S4). The DelVG lengths of the corresponding datasets were combined and the difference of the distributions was assessed using Wilcox–Mann–Whitney *U* (Supplementary Table S5) and Cliff’s delta [50] (Fig. 2B). Indeed, we found differences of the distributions in the DelVG lengths depending on the host system. In particular, the lengths of DelVGs from *in vitro* experiments are, on average, 232 nt shorter than those derived from human samples (Fig. 2B). Cliff’s delta is smaller for the mouse datasets (PA: −0.19, PB1: −0.21, and PB2: −0.24) compared with the human datasets (PA: −0.29, PB1: −0.39, and PB2: −0.46). There is also a difference between human- and mouse-derived *in vivo* datasets, but the effect is small for PB1 (−0.27) and PB2 (−0.25), and negligible for PA (−0.10). It should be noted that five out of six human datasets are from IBV and that the DelVGs from the IBV datasets, independent of the host system, are on average 121 nt longer than DelVGs from all analyzed IAV datasets.

To confirm that the host system mainly drives our observation, we directly compared different subsets of the data, considering the influenza types and host systems (Supplementary Fig. S5A–F). For a combined analysis of these factors, the Scheirer–Ray–Hare test was performed, which is the nonparametric pendant to the two-way ANOVA [43]. We excluded the mouse datasets from this analysis, because no such datasets were available for IBV (Supplementary Table S4). Because the test was performed for the pooled data (eight segments) and for each of the three polymerase segments independently we employed multiple testing corrections by Bonferroni (*n*= 4, α = 0.0125). We find significant *P*-values for the hypothesis that the host system impacts the DelVG length for the pooled dataset (H = 6.91, *P* = 0.0086) as well as the PB2 (H = 7.47, *P* = 0.0063) and PA (H = 9.3, *P* = 0.0023) segments (Supplementary Table S6). For the influenza type, we find no significant difference for the pooled data (H = 1.93, *P* = 0.1651) or the three polymerase segments (PB2: H = 3.55, *P* = 0.060; PB1: H = 0.8, *P* = 0.37; and PA: H = 3.18, *P* = 0.074). However, note that we find a significant *P*-value for the interaction of the influenza virus type with the host system for the pooled data (H = 7.16, *P* = 0.0074) and the PB1 segment (H = 10.33, *P* = 0.0013). These results of the Scheirer–Ray–Hare test strengthen our speculation that the host system influences the DelVG sequence length (Supplementary Table S6). Note that due to the data imbalance in the number of DelVGs from different influenza types and host systems (Supplementary Table S4), our findings on the impact of both variables on DelVG length would benefit from validation in independent experiments.

### Long DelVGs may provide new insights on DelVG formation

Besides the DelVGs with a short sequence, DelVGs with a sequence length around 2000 nt are found in all of the datasets for the PB2 segment (Fig. 2A). This contradicts the general observation that deletions span between the termini of the parental sequence [28]. To investigate the occurrence of these long DelVGs in more detail, the fraction of DelVGs with a length >85% of the full sequence of the respective parental genome segment was determined (Supplementary Table S7). While the length of long DelVGs’ deletions can be up to 200 nt, 84.6% of long DelVGs have a deletion between 20 and 100 nt. In contrast to short DelVGs with an approximate symmetric deletion site [24, 28, 29], long DelVG deletions occur mostly either in regions at the 5′- or 3′-ends of the sequence (Fig. 2C and Supplementary Fig. S6). We observed at least 0.7% and up to 26.4% long DelVGs in the analyzed DelVG populations (Supplementary Table S7). Hence, we are convinced that long DelVGs are a considerable experimental observation and its origin needs to be elucidated. We evaluated if the long DelVGs differ from short DelVGs with respect to frame shifts, direct repeats, and nucleotide enrichment, but observed no significant difference (data not shown).

It is most commonly proposed that the formation of short DelVGs is caused by a template switch of the viral polymerase (reviewed by [3, 7, 11, 14, 51]). For this, the polymerase detaches from its current template and continues replication further downstream of the sequence, a process that is facilitated by the secondary structure of the viral genome segments that adapt a rod-like shape which brings distant parts of the sequence in physical proximity, e.g. in the minor groove of the segment’s helical structure (Fig. 2D, green arrow). Similarly, long DelVGs could be formed by a template switch. However, for long DelVG formation, the polymerase may re-attach closer to its current position and, thus, truncate a significantly smaller portion of the template sequence. We hypothesize that the template switch may also occur within the major grooves of the segment’s helical structure (Fig. 2D, pink arrow). This hypothesis is partially supported by a study on the structure of influenza virus genomes that estimated the spacing between two helical turns to be 17 nm [52]. While 17 nm, in theory, corresponds to 158 nt, the modeling of electron tomography measurements suggests that 17 nm corresponds to 284 nt in genomic sequence length. Hence, based on the either theoretical or empirical approach, the helical arc length is estimated between 158 and 284 nt, respectively [52]. Considering the dynamics of the viral genome structures and the resulting uncertainty in their quantitative assessment, template switches spanning the major groove are a potential explanation for short deletions with up to 200 nt. However, a thorough validation would require a better understanding of the viral ribonucleoprotein (vRNP) structure and replication mechanism. Likewise, the previously proposed “looping-out” model [11] could provide a potential explanation for long DelVG formation.

### The 5′- and 3′-end of the DelVGs are different between IAV and IBV

**Figure 3.**
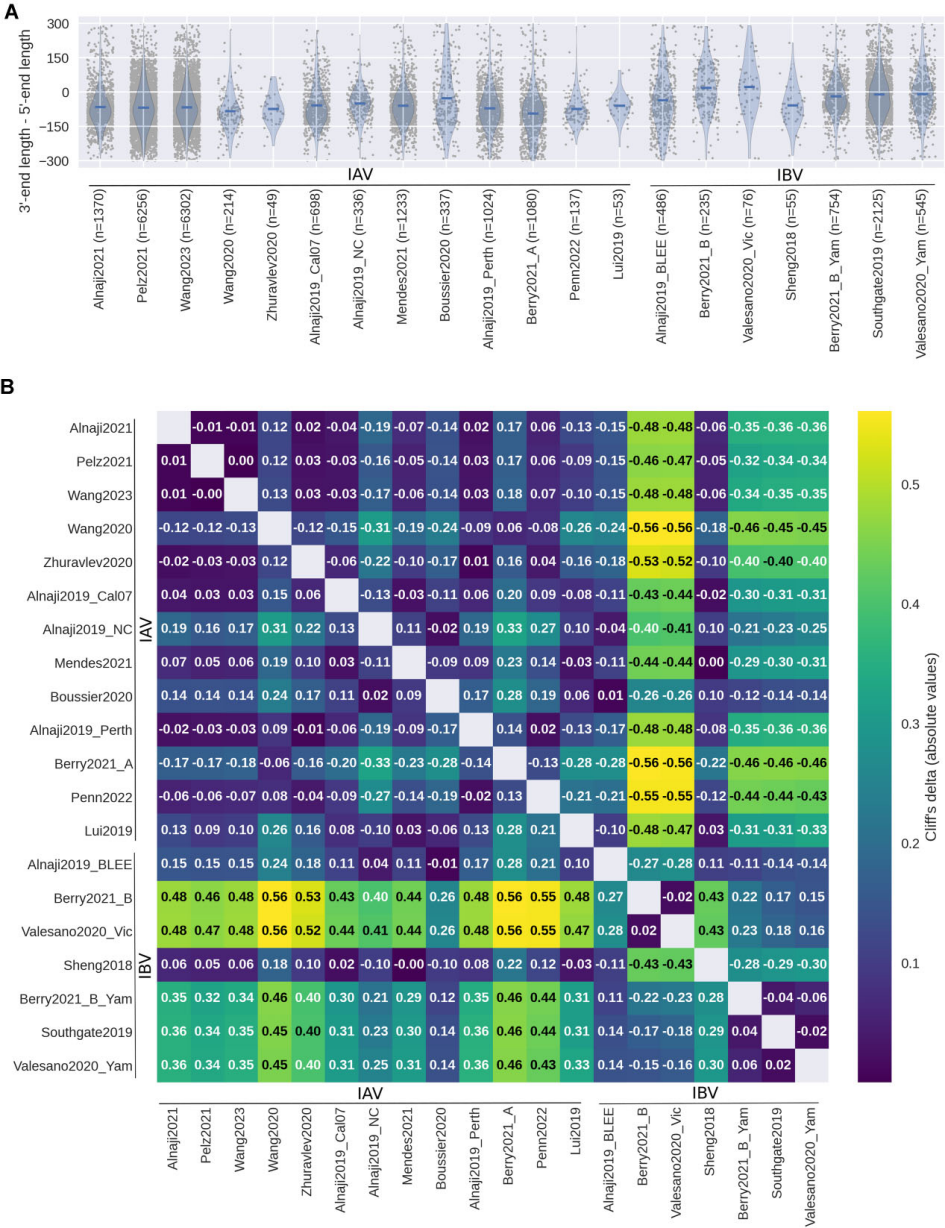
Difference of 3′-end and 5′-end sequence lengths. (**A**) Difference of 5′- and 3′-end lengths of the DelVGs. The mean of each dataset is depicted in the violin plots by a blue bar. Only DelVGs with a difference between −300 and 300 were considered to exclude long DelVGs. (**B**) Pairwise comparison of the dataset distributions using Wilcox–Mann–Whitney *U* and Cliff’s delta for estimating the effect size. The absolute values of Cliff’s delta were used for coloring. The closer Cliff’s delta is to 1 the more the dataset on the *y*-axis dominates the dataset on the *x*-axis. Dominance means that the differences of the 5′- and 3′-ends are more often larger in the *y*-axis dataset than in the *x*-axis dataset. Absolute effect sizes are considered negligible (<0.15), small (0.15–0.32), medium (0.33–0.46), or large (>0.46).

To further examine DelVG characteristics, we compared the length of the retained sequences at the 3′- and 5′-end. The length of the 5′-end was subtracted from the 3′-end to compare the relation of the lengths. DelVGs with a difference smaller than −300 or bigger than 300 were considered outliers and removed from the analysis. For all IAV strains, the mean length of sequences on the 5′-end of the DelVG is, on average, 66 nt longer than on the 3′-end (Fig. 3A). In contrast, 3′- and 5′-ends of DelVGs originating from IBV strains are, with an average length difference of 12 nt, closer to a symmetrical deletion pattern (Supplementary Table S8, row 1). The difference between the two values is smaller when considering the host system, comparing the *in vitro* (62 nt) and human (24 nt) datasets (Supplementary Table S8, row 2). To evaluate this finding, it was tested if the datasets have the same distribution of their 3′- and 5′-end lengths. Therefore, Wilcoxon–Mann–Whitney *U*-test was performed (data not shown) and Cliff’s delta was calculated for each dataset combination individually. Overall, Cliff’s delta is closer to zero when comparing datasets from the same influenza type and reaches absolute values up to 0.56 when comparing IAV and IBV datasets. This implies that there is a noticeable difference between the influenza types (Fig. 3B). In the resulting matrix, two clusters can be observed, one including IAV datasets (upper left corner) and the second one including IBV datasets (lower right corner). DelVGs obtained from Boussier2020 [27], Alnaji2019_BLEE [28], and Sheng2018 [36] oppose this observation with means that tend towards the means of the other influenza type (Fig. 3A). To verify whether this difference is observed due to the influenza virus type and not the host system, the distributions of the differences of the 3′- and 5′-end across various data splits (Supplementary Table S8) were visualized (Supplementary Fig. S5G–L) and additionally analyzed utilizing the Scheirer–Ray–Hare test (Supplementary Table S6). We excluded the mouse datasets from this analysis, because no such datasets are available for IBV (Supplementary Table S4). Again the multiple testing correction by Bonferroni was applied (*n*= 4, α = 0.0125). The results of the Scheirer–Ray–Hare test indicate a significant effect for the influenza type for the pooled data (H = 9.45, *P* = 0.0021) and two of the three polymerase segments (PB1: H = 7.71, *P* = 0.0055; PA: H = 6.65, *P* = 0.0099) and no effect of the host system. The interaction of the two parameters (influenza type and host system) possesses no significant effect after applying correction for multiple testing by Bonferroni. This shows that the influenza type has indeed an impact on the 3′- and 5′-sequence lengths. Note that most of the calculated *P*-values are close to the selected significance level α and the underlying imbalanced distribution of the data between the parameters, i.e. number of data points *in vitro* versus human (Supplementary Table S4 and S8), could bias the results of the test. Therefore, validation of our findings in independent experiments is needed.

### Longer than expected direct repeats occur in all datasets except for A/California/07/2009

**Figure 4.**
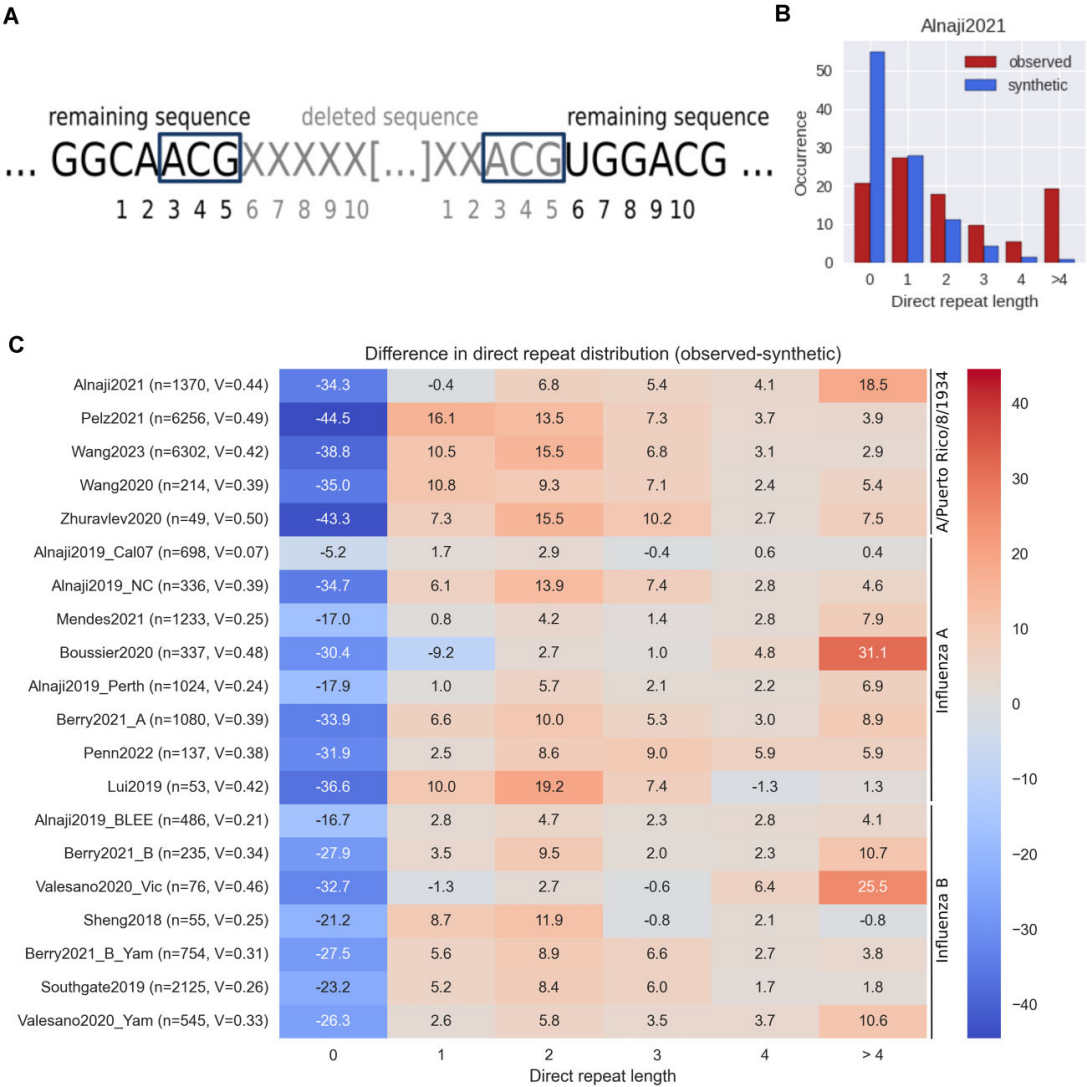
Evaluation of differences in the direct repeat lengths across all datasets. (**A**) Deletion site for a DelVG with a direct repeat of length 3. The sequences before the start and the end of the deletion site are compared to identify a direct repeat (blue box). Nucleotides that are deleted are colored in gray and nucleotides that remain in the DelVG are colored in black. (**B**) Distribution of the direct repeat lengths for Alnaji2021 [29] in percent. The occurrence of each class was compared with randomly generated synthetic data. (**C**) Direct comparison of the distribution of the direct repeat lengths for all datasets. The results were compared by calculating the difference of the percentages between the observed and the synthetic data. Statistical effects were tested on absolute counts with one-way chi-squared test and calculating Cramer’s V [44] as effect size. (Cramer’s V effect size [46]: V < 0.1 = negligible; 0.1 < V < 0.2 = weak; 0.2 < V < 0.4 = moderate; 0.4 < V < 0.6 = relatively strong; 0.6 < V < 0.8 = strong; 0.8 < V < 1.0 = very strong).

The predominant explanation for the emergence of DelVGs is the polymerase template switch, which is possibly driven by specific nucleotide patterns. Publications by Saira *et al.* [25] and Lui *et al.* [26] indicate that a short, repeated nucleotide sequence exists at the start and end position of the deletion site, referred to as direct (sequence) repeat [27]. It is hypothesized that the direct repeats are mediating the template switch of the viral polymerase [25, 26, 29]. A direct repeat is defined by the number of nucleotides that are identical between the sequence before the start and end of the deletion site (Fig. 4A). For each dataset, the direct repeats were calculated as done by Alnaji *et al.* [29], with the addition that we pooled over all segments and grouped in six categories (0, 1, 2, 3, 4, and >4). We generated random synthetic data (see the "Random sampling approach to generate synthetic datasets" section) to compare the distribution of the direct repeat lengths (Fig. 4B). Overall, DelVGs containing a direct repeat occur more often compared with randomly sampled synthetic data (Fig. 4C). By applying a one-way chi-squared test and calculating Cramer’s V [44] as effect size, we could validate that the overall distribution of direct repeats is not the same as the distribution of the direct repeats in synthetic data. Only for the Alnaji2019_Cal07 [28] dataset, the distributions align well, represented by a negligible effect size (V = 0.07), which is in agreement to the original paper [28]. This observation was further confirmed by comparing the direct repeat lengths of the individual datasets to the mean distribution of all datasets (Supplementary Fig. S7). Here, we observed that A/California/07/2009 DelVGs tend to have more often no direct repeat sequences (+24.3 percentage points) at the deletion site compared with other datasets. Additionally, the comparison to the mean of all datasets revealed that the Boussier2020 [27] and Valesano2020_Vic [34] datasets include more DelVGs with direct repeat lengths >4 compared with the other datasets. Since it is hypothesized that the direct repeats are associated with the formation of DelVGs mediated by the polymerase template switch [27], we compared the polymerase protein sequences of all investigated virus strains. In particular, we examined if mutations on the polymerase-encoding segments can be found exclusively for A/California/07/2009, which may also influence the polymerase function. A multiple sequence alignment (MSA) was conducted for the reference sequences of the three polymerase segments using MAFFT [53] (mafft.cbrc.jp) with default parameters. By the MSA, we identified the mutation K627E on segment PB2 in the A/California/07/2009 strain (Supplementary Fig. S8). This position is known to be associated with the binding of nucleoproteins and polymerase activity [54].

### The nucleotide pair “UA” is enriched around the deletion sites

**Figure 5.**
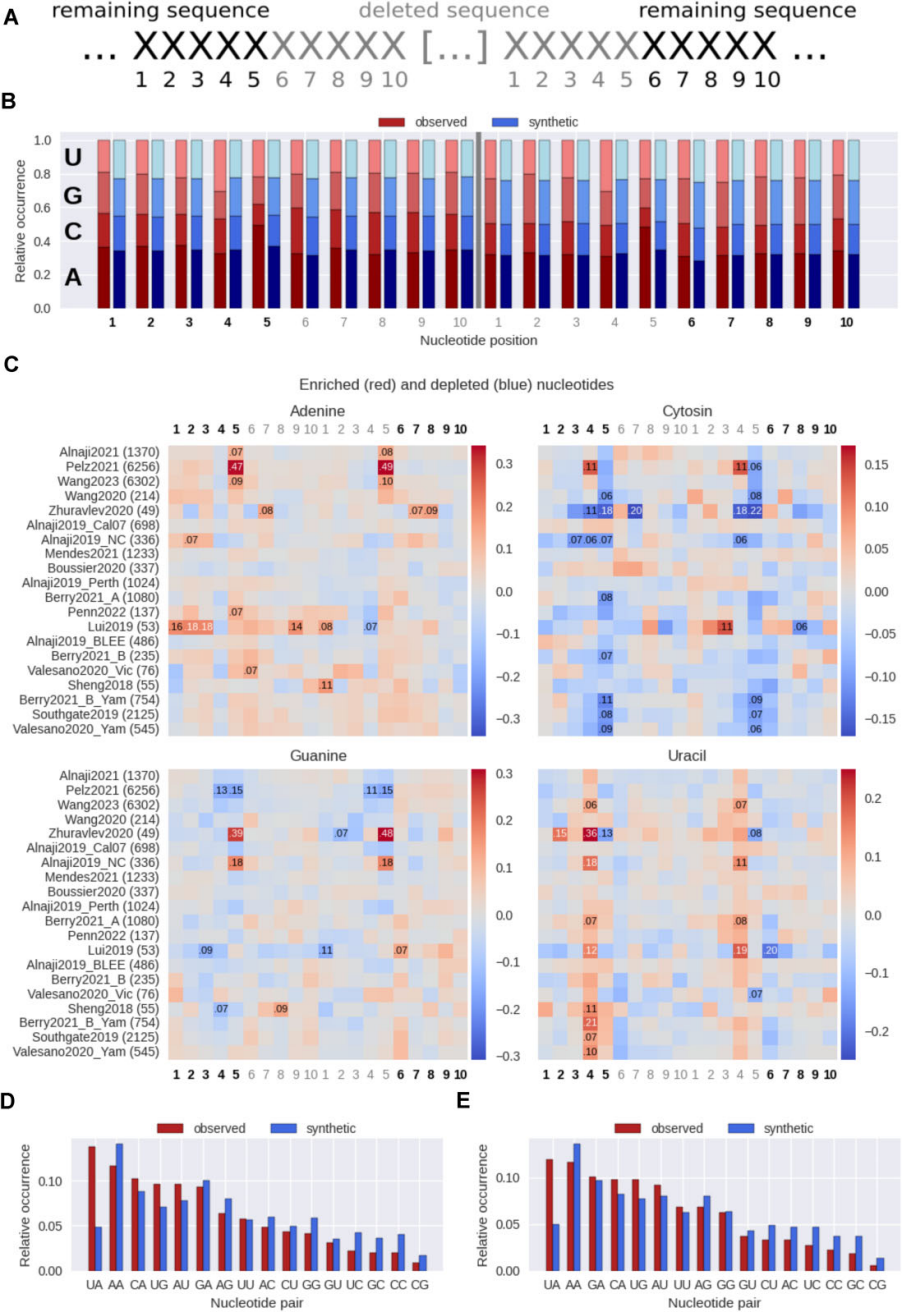
Nucleotide enrichment at the deletion site. (**A**) Overview of the considered nucleotides around the deletion site. Two areas of length 10 were defined, one at the start and one at the end of the deletion site. The nucleotides that remain in the DelVG sequence are colored in black, deleted nucleotides are in gray. (**B**) Relative occurrence of the nucleotides around the deletion site for Alnaji2021 [29]. The observed frequencies are given on the left of each position (red) and the frequencies of the synthetic data are given on the right (blue). The nucleotide positions in gray are part of the deleted sequence, while black ones remain in the DelVG. (**C**) Comparison of observed and synthetic frequency of each nucleotide at indicated positions around the deletion site. To compare the values the difference between the observed and synthetic frequency was calculated. To test if the observed and synthetic frequency per nucleotide position are originating from the same distribution, the Kruskal–Wallis test was used. For positions with significant *P*-value (*P* < 0.05), the effect size η^2^ was calculated and reported for positions with at least a medium effect (η^2^ ≥ 0.06), where η^2^ ≥ 0.14 denotes a large effect [63]. (**D**, **E**) The observed relative occurrence of the nucleotide pairs (left bar, red) is depicted together with the data from randomly sampled synthetic datasets (right bar, blue). The nucleotide pairs are sorted by their occurrence in decreasing order in the observed data. The nucleotide pairs at the start of the deletion site are shown in panel (D), while the nucleotide pairs at the end are shown in panel (E).

The occurrence of specific nucleotides, or short motifs, at the deletion site could induce the polymerase template switch. Therefore, in addition to direct repeats, the nucleotides surrounding the deletion site are considered as relevant features, e.g. for DelVG formation. For instance, Boussier *et al.* [27] and Alnaji *et al.* [29] found an enrichment of adenine and uracil in direct repeat sequences or at the deletion site, respectively. The occurrence of the four nucleotides around the deletion site was counted for all datasets (Fig. 5A). For Alnaji2021 [29], adenine is enriched at the position 5 at the start and the end of the deletion (Fig. 5B). For each dataset individually, adenine and uracil are more often enriched (A: 18, U: 14) than depleted (A: 1, U: 4) compared with random synthetic data (Fig. 5C). In contrast to that, cytosine and guanine are more often depleted (C: 21, G: 8) than enriched (C: 3, G: 6). The enrichment and depletion accumulates at the deletion site start and end point. The comparison with the synthetic dataset gives a perspective on possible mechanisms on the emergence of DelVGs. An additional comparison to the mean over all datasets provides an insight on dataset-specific differences. Overall, we found less significantly enriched nucleotides upon comparison to the mean of all datasets, which supports the presence of common nucleotide enrichment patterns across all datasets. However, note that highly enriched nucleotides at very prominent positions, e.g. in Pelz2021, positions 5, were detected by both analysis approaches, i.e. comparing to either mean or to the synthetic data, respectively (Supplementary Fig. S9). To elaborate further on nucleotide enrichment and link it to the direct repeats, the frequency of the nucleotide pairs at position 4 and 5 of the start and end site of the deletion was calculated for each dataset. These two positions are relevant for the direct repeat analysis. The nucleotide pair “UA” is the most frequent one across all datasets for the start and end site (Fig. 5D and E). For this, no difference between IAV and IBV strains was evident (data not shown). The nucleotide pair “UA” appears approximately twice as much as expected in the synthetic datasets. The most frequent nucleotide pair for each dataset can be seen in the supplement (Supplementary Table S9). In the synthetic data, the nucleotide pair “AA” is the most frequent one at the start and end of the deletion site in all datasets. It seems reasonable that the motif “AA” is observed most frequently, because adenine occurs most frequently in the RNA sequences of the influenza virus genome as stated by Boussier *et al.* [27]. For the hot spot areas that were defined to generate the synthetic data (see the "Random sampling approach to generate synthetic datasets" section), the occurrence of adenine ranges from 27.6% for segment M of A/Puerto Rico/8/1934 to 42.2% for segment M of B/Brisbane/60/2008. The probability of two adenines occurring next to each other and forming an “AA” nucleotide pair would therefore be between 7.6% and 17.8%. All observations in the synthetic data are in this range, except for the starting site of Sheng2018 [36], Berry2021_B_Yam [33], and Southgate2019 [35]. The difference between the most frequent nucleotide pair being “UA” in the original data and “AA” in the random data suggests that this nucleotide pair is potentially important for DelVG formation.

### Identification of top DelVGs for A/Puerto Rico/8/1934

To identify most abundant DelVGs, we intersected the DelVGs of all processed A/Puerto Rico/8/1934 datasets and ranked them according to their abundance. Of note, a high abundance of DelVGs may have different biological implications. For instance, DelVGs might occur more frequently due to conserved mechanisms of DelVG formation and/or selection. Recently, it was proposed that a high abundance of DelVGs directly correlates to their antiviral potential, assessed by virus load reduction experiments [24]. Hence, we may assume that our ranking to obtain top DelVGs, i.e. highly abundant DelVGs, can be used to pre-select the most promising candidates for future development of antiviral treatments.

**Figure 6.**
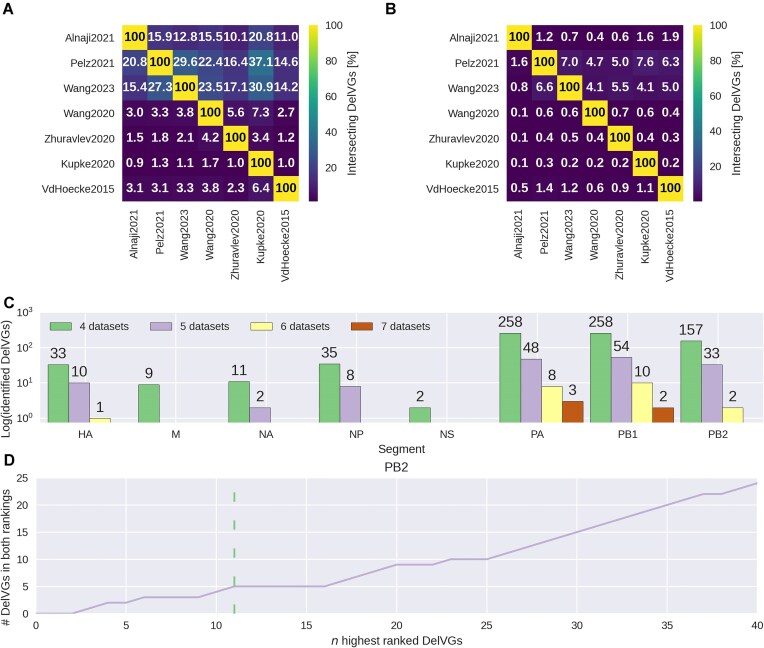
Identification of relevant DelVG candidates by ranking candidates of seven A/Puerto Rico/8/1934 datasets. (**A**) Intersection matrix of the seven considered datasets. Each number indicates the percentage of DelVGs of the dataset on the *x*-axis that are found in both datasets. (**B**) Intersection matrix of the randomly generated data. (**C**) Number of identified DelVGs that occur in multiple datasets per segment. The color of the bars indicates the number of datasets (4 to 7 from left to right) that include the same DelVG. (**D**) Detection of top PB2 DelVGs based on the two rankings (sum, mean). With increasing *n* the number of DelVGs that are ranked in both rankings (sum, mean) at position *n* or higher increases. The green dashed line (*x* = 11) indicates the threshold used to select the final top DelVG candidates.

The intersection, which is calculated on junction identity, between the dataset pairs ranges from 0.9% to 37.1%, while about half of the pairwise intersections are <5% (Fig. 6A). The larger pairwise intersections (>10% intersecting DelVGs) include at least one of the larger datasets (Pelz2021 [24] *n*= 33 595, Wang2023 [37] *n*= 30 976, and Alnaji2021 [29] *n*= 25 662). To verify if the found DelVGs are relevant, we conducted the same analysis, but using synthetic datasets (see the "Random sampling approach to generate synthetic datasets" section) of the same sizes. The overlap between these synthetic datasets ranges from 0.1% to 7.6% and is therefore smaller than for the experimental ones (Fig. 6B). Hence, the DelVG candidates that occur in multiple of the experimental datasets are found more frequently than by chance.

For all eight segments, DelVGs are identified that occur in at least four datasets (Fig. 6C). More DelVGs are present for the three polymerase segments, because they provide overall more DelVGs (Fig. 1A). The underlying distribution of the identified DelVGs per segment and the DelVG occurrence over all datasets per segment was compared with a chi-squared test and Cramer’s V [44] and showed a weak association (V = 0.19). Hence, the higher abundance in intersecting DelVG candidates is in accordance with the abundance across all datasets. Five identified DelVGs occur in all seven datasets (PA: 3 and PB1: 2). For PB2, two DelVGs are observed in six of the datasets. The percentile of the NGS count of each DelVG was estimated for each dataset. Using these percentiles, the DelVGs were ranked by calculating the sum and the mean of the percentiles. This ranking was performed for each polymerase segment independently. The final DelVGs were selected by intersecting the two rankings (sum and mean). The PB2 DelVGs that occur in both rankings at rank *n* or higher were determined for an increasing *n* (Fig. 6D). By this, we identified five PB2 DelVGs that occur in both rankings at position 11 or higher. The threshold 11 was selected as it provides a good separation to the next DelVG, which would be included at *n* = 16. The identified PB2 DelVGs are also present in Pelz2021 [24], which provides a longitudinal sequencing analysis for a continuous infection study. Hence, the study allows evaluating the enrichment of individual DelVG candidates over a time-course of 3 weeks [24]. In particular, Pelz *et al.* defined candidates enriched over time as “gain” and those that were depleted over time as “loss” candidates. Besides, the study also allowed to track the enrichment of *de novo* DelVG candidates that were not contained in the seed virus. While Pelz *et al.* focused on those *de novo* candidates that were enriched over time (“*de novo* gain”), we also determined those *de novo* DelVG candidates that were depleted before the end of the experiment (“*de novo* loss”). Only one of the five identified DelVGs, PB2_177_2141, is a *de novo* loss candidate. The other four are either labeled as gain (PB2_109_2152) or *de novo* gain (PB2_163_2139, PB2_163_2152, and PB2_191_2048). The evaluation of the identified DelVG candidates for all polymerase segments is presented in the supplementary material (Supplementary Table S10).

To contextualize the identified candidates within the meta-analysis, we analyzed the candidates with respect to the same features, such as DelVG length, frame shift, and nucleotide enrichment. Most identified candidates have a DelVG length ranging from 290 to 485 nt, except for two candidates (PA_139_169 and PB1_54_98), with a DelVG sequence length >2200 nt. The identification of these two candidates underline our hypothesis that long DelVGs are common in DelVG populations. Furthermore, all eleven candidates display out-of-frame deletions and contain a direct repeat of at least length 1, aligning with the findings of the meta-analysis (Figs 1B and 4C). Likewise, 8 of the 11 candidates possess the two-nucleotide motif “UA” at the start, or start and end position of their deletion site in agreement to our previous observations (Supplementary Table S9). Additionally, we verified the presence of the identified candidates both *in vivo* and *in vitro*. A candidate for medical application will probably be produced *in vitro* and applied *in vivo*. Therefore, it is beneficial for such candidates to propagate well in both environments. Notably, all identified candidates are present in Wang2023 [37], which is the only *in vivo* dataset for A/Puerto Rico/8/1934 in our study. All features that were analyzed for the eleven identified candidates can be found in the supplementary material (Supplementary Table S10).

## Discussion

DelVGs have been investigated in many studies in the last decades. The studies use different analysis methods to identify and evaluate the DelVGs. For instance, different features are utilized to describe the DelVG populations, which challenges direct comparison of the study results. Therefore, we performed a meta-analysis of 20 IAV and IBV datasets. We analyzed the selected datasets by the same DelVG detection pipeline [28] to ensure the same preprocessing and make the results more comparable. The datasets were analyzed for their features, such as DelVG length, direct repeats, and nucleotide enrichment. This meta-analysis enabled us to compare datasets of different sources more accurately and identify the impact of factors, such as the host system or virus strain.

In the past, mainly short DelVGs, with a sequence length of around 500 nt were investigated since they make up the biggest fraction of identified DelVGs [18, 22, 24, 25]. We identified the occurrence of long DelVGs, which contain at least 85% of the original sequence, in all analyzed datasets. The long DelVGs are mentioned in the literature, but were not investigated in more detail since the focus is usually on the short DelVGs [18, 24, 27, 28]. Since we observed long DelVGs to at least some extent in all the 20 datasets, we argue that they should not be neglected. The presence of long DelVGs introduces the possibility that the template switch, which is the most frequently proposed mechanism of DelVG formation, may also lead to the truncation of shorter sequence parts of 20–100 nt. We hypothesize that both, long and short, deletions arise due to the template switch, which either occurs in the minor or major groove of the helical viral genome structure, respectively. Alternatively, the recently proposed “looping-out” model provides an additional explanation for short-sequence truncations [11].

We provide indication that DelVGs observed in human *in vivo* samples of IAV are longer than for *in vitro* systems (Fig. 2B). Therefore, we assume that the host system of the experiment has an influence on the DelVG length. Duhaut and Dimmock (1998) found that the terminal 3′- and 5′-ends are longer in mouse lungs than in an inoculum. They argue that “[…] some selection may be occurring in the lung” [18]. Alnaji *et al.* demonstrated a difference of lengths between intra- and extracellular *in vitro* DelVGs [29]. The intracellular DelVGs in their experiments are overall shorter than extracellular DelVGs. They argue that the packaging mechanism is responsible for that selection. Moreover, a recent study by Li *et al.* suggests a higher prevalence of copy-back defective influenza viral genomes compared with DelVGs in clinical human samples compared to *in vitro* samples [55]. These observations highlight the impact of the host system on the generation and selection of the DelVGs and thus, on the shaping of DelVG populations. Likewise, the differences in DelVG populations between *in vivo* and *in vitro* experiments may also have implications for the development of DelVG-based treatments and should be taken into consideration. Likely, particles harboring the DelVG will be produced in cell culture-based production systems *in vitro*, and applied *in vivo*. Hence, acknowledging differences between the two systems is crucial for successful treatment development.

In our meta-analysis, we could demonstrate that the 5′-end and 3′-end retained in the deleted sequence are of similar length for IBV. For IAV, the 3′-ends are on average longer than the 5′-ends (Fig. 3A), which is a finding supported by several previous studies [18, 24, 56]. The difference between IAV and IBV for the 3′- and 5′-ends is to our knowledge not yet described in literature. We could speculate that the 3′ end of IAVs contains elements relevant for DelVG formation and/or replication, indicating that it is potentially more crucial for IAV DelVGs. Possibly, the retained 3′ elements might be also involved in a IAV-specific packaging process. Thus far, the length of the packaging signal and noncoding region reported for IAV is of similar length for the 3′- and 5′-end (reviewed by Breen *et al.* [57]). Therefore, our finding might point to a potential, yet unknown, packaging mechanism involving elements of the 3′-end. Experiments specifically designated to this question could provide, both, an improved understanding of the DelVG as well as the wild-type virus life cycle. Other aspects, such as the secondary structure, could be an additional factor, as previously stated by Duhaut and Dimmock [56]. The difference of the 3′- and 5′-end lengths shows that DelVGs from different types of influenza viruses may vary. Moreover, there may be other, yet unknown differences between the influenza virus types. Although it was previously shown that IAV DelVGs protect IBV-infected mice [58], our finding could imply that it is crucial to account for the influenza type when selecting the most effective treatment in medical applications.

**Figure 7.**
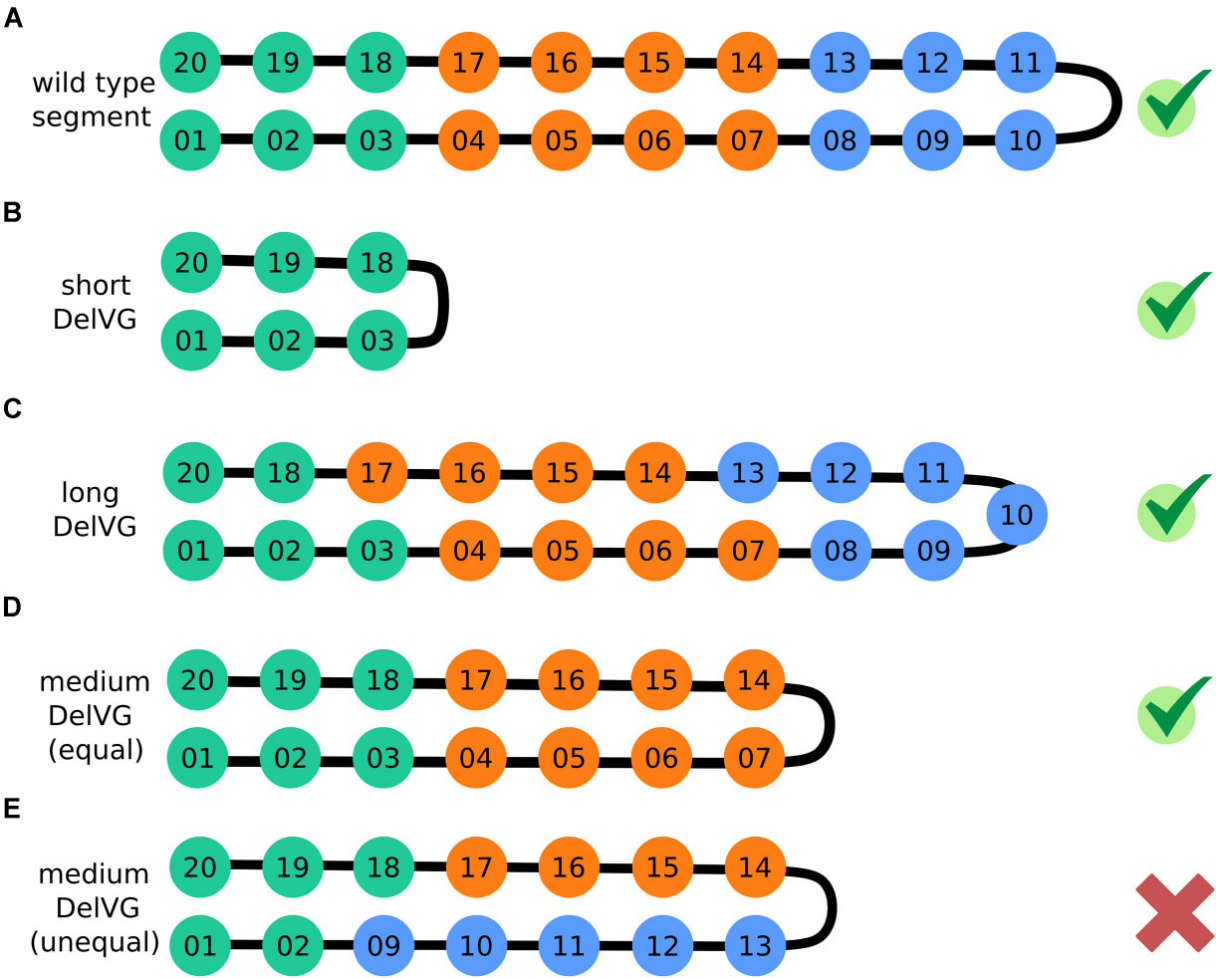
Impact of differently sized single deletion DelVGs on the assembly of the vRNP. (**A**) Wild-type vRNP. The whole sequence is represented in 20 subsequences. The numbering of the subsequences indicate their location in the vRNP (3′- and 5′-end: 1-3 & 18-20, green; middle: 4-7 & 14-17, orange; vRNP loop region: 8-13, blue). (**B**) Short DelVG with a long deletion site. The subsequences 4–17 are deleted. The remaining subsequences are arranged in the same way as in the wild-type vRNP. (**C**) Long DelVG with deleted subsequence 19. Because of the short deletion site, most subsequences are located next to each other as in the wild-type vRNP. (**D**) Medium sized DelVG with equal lengths of the 3′- and 5′-end sequences. The subsequences 8–13 are deleted, and the remaining subsequences are arranged in the same way as in the wild-type vRNP. (**E**) Medium sized DelVG with unequal lengths of the 3′- and 5′-end sequences. The subsequences 3–8 are deleted. This deletion leads to a shift of the subsequences and results in a different arrangement compared with the wild-type vRNP.

In our analyses, we observed long DelVGs that retain >85% of the wild-type sequence next to DelVGs of short and medium length (Fig. 2A). Additionally, we could demonstrate that for the short and medium DelVGs the 3′- and 5′-ends of the sequence are about the same length (Fig. 3), which stems from symmetric deletion sites (Supplementary Fig. S6). This symmetry is not applicable for the long DelVGs, because of their short deletion. We assume that this different symmetry can be explained by the shape of the vRNP (Fig. 7). If the deletion retains two ends of similar length, as it is the case for the short and medium DelVGs, the same subsequences are in proximity in the vRNP as in the wild type (Fig. 7B and D). For the long DelVG, the same subsequences as in the wild type are close to each other, because only a few nucleotides are deleted (Fig. 7C). For a medium sized DelVG that contains unequal lengths at the 3′- and 5′-end, the alignment of the subsequences around the loop area is different than in the wild-type vRNP (Fig. 7E). This variation in the location of the subsequences could lead to a vRNP, which is either not that stable, cannot be packaged as good, or is due to some other reason not as viable as the wild-type vRNP. The presented idea aligns with the observation that conserving the secondary structure in dengue virus DelVGs is crucial for replication [59].

It is hypothesized that direct repeats play a role for DelVG formation by inducing the template switch of the polymerase [7, 25, 27]. Indeed, we also found that direct repeats occur more often in experimental DelVGs compared with random synthetic DelVGs (Fig. 4). Only Alnaji2019_Cal [28] had no significant difference of the direct repeats compared with the synthetic data. This dataset is from the same experiment based on which Alnaji *et al.* drew their conclusion that direct repeats are not associated with junction formation [28]. By an MSA, we could show that A/California/07/2009 is the only strain with the mutation K627E on the PB2 sequence. The amino acid E627 is known to be associated with the binding of nucleoproteins and polymerase activity [54]. The activity is promoted by the positive charge at position 627 and 630. Vasilijevic *et al.* showed that a single mutation (D529N in the PA segment) leads to a reduction in DelVG numbers [60]. This shows that a single mutation in one of the polymerase subunits can have a considerable impact on the formation of DelVGs. We therefore assume that the identified mutation K627E could be the reason for the reduced number of direct repeats in A/California/07/2009. Only one dataset was available for A/California/07/2009 in our meta-analysis, which limits the generalizability of our findings. To verify the impact of the mutation K627E, it would be necessary to conduct more experiments with the A/California/07/2009 strain and assess if the resulting direct repeat lengths are reproducible. Alternatively, it would be also possible to induce this mutation in another strain, e.g. A/Puerto Rico/8/1934, and evaluate its impact on the direct repeat distribution.

The enrichment of adenine and uracil around the deletion site was already described in literature [29]. We could partially verify this observation, but we also show that this enrichment is not consistent throughout all datasets. This demonstrates that DelVG occurrence is at least partially a result of stochasticity inherent in biological processes, such as genome replication. This stochasticity can also be observed when comparing the DelVGs between replicates in the same experiment [29]. When analyzing the nucleotides around the deletion sites, the conducted method is crucial for the results. For example, Boussier *et al.* found no enrichment of nucleotides when testing on a group of 20 nt positions (10 upstream and 10 downstream of the deletion site) [27]. But they found a significant enrichment of A and U in the direct repeat sequences. Alnaji *et al.* found an enrichment of nucleotides, when considering each position individually [29]. This individual consideration was also performed in this meta-analysis. In our opinion, grouping over multiple positions might be disadvantageous, because the enrichment of nucleobases at specific positions is masked by taking into account too many nucleotide positions simultaneously.

As an attempt to perform a joint analysis of direct repeats with the enrichment of nucleotides, the most frequent nucleotide pair at the start and end of the deletion site was determined for each dataset. The nucleotide pair “UA” was observed most frequently for the start and end of the deletion (Fig. 5D and E). It also differs from randomly generated synthetic data, where the nucleotide pair “AA” is observed most frequently in all datasets (Supplementary Table S9). Hence, the enrichment of “UA” is biologically relevant and not a result of random deletions. With this nucleotide pair, we provide a correlation of the direct repeats and nucleotide enrichment and propose a possibly important nucleotide motif for DelVGs. Furthermore, the relevance of the nucleotide pair “UA”, which is enriched at the deletion site, is supported by other studies that show an enrichment of adenine and uracil in direct repeats [27] or at the 5′ deletion site [29]. Because of that, we suggest that the nucleotide pair “UA” could be an inducer of the template switch, which, however, requires further experimental validation.

Previously Pelz *et al.* [24] demonstrated that DelVGs which enrich over time reduce infectious virus loads to a higher extent compared with other DelVG candidates, i.e. highly abundant DelVGs are potentially highly antiviral. To identify potentially interesting candidates, we searched for DelVGs that are present frequently and highly abundant in multiple datasets of A/Puerto Rico/8/1934. The intersection between the dataset pairs is higher compared with random synthetic data (Fig. 6A and B). Hence, the intersected DelVGs were unlikely to be identified by chance. Given their prevalence across independent experiments, we assume that they occur due to one or multiple common mechanisms. These mechanisms may shape the DelVG populations and can have an impact during formation and/or selection of the DelVGs. Notably, the seven datasets originate from diverse experimental setups, e.g. different host systems including cell lines, and mice, which further underlines the relevance of the intersected DelVG candidates. In particular, we can rule out that the intersected candidates stem from the same seed virus material. Thus, when repeating the presented approach based on experiments from just one laboratory, it is crucial to evaluate that the identified DelVGs are no in-house artifact.

We prioritized 11 DelVGs for the three polymerase-encoding segments (PB2: 5, PB1: 3, and PA: 3) by using the mean and sum of the percentiles of their abundances as scores. The identified DelVGs were assessed using the same features as in the meta-analysis (Supplementary Table S10). Important patterns, such as direct repeats and the nucleotide pair “UA” at the deletion site, were also identified for the intersecting DelVGs, highlighting their significance. Although computational identification of these DelVGs is feasible, it is yet not possible to verify their antiviral potential computationally. Additionally, a high abundance of DelVG candidates may also correlate with yet unknown features of DelVGs, which may or may not be related to antiviral activity. To elucidate the impact of our top 11 DelVGs on virus replication, experimental assessment of virus load reduction and host response need to be followed-up in future studies.

The meta-analysis comes with some limitations that we also want to elaborate on in the end. One limitation is that we focused only on single-deletion DelVGs. In a next step, the presented analysis could be expanded to consider other types of DelVGs, such as those with multiple deletions, or copy-back [55] DelVGs. Potentially, such extended meta-analysis would allow drawing a more comprehensive picture of the mechanisms behind DelVG formation and replication. A second limitation is that for experiments that provide time-resolved sequencing data or replicates, identified DelVGs were pooled. This allowed us to jointly compare a broader bandwidth of datasets, at the expense of those datasets’ complexity. For the datasets with time series data, it would be interesting to elucidate in more detail the changes in the composition of the DelVG population over time. However, since the time spans of the individual experiments can range from hours [29] to weeks [24], it might be challenging to compare multiple time-series experiments in a meaningful way. Additionally, the cultivation methods of the time-series experiments, such as independent experiments with different end points [29, 36] as well as batch-wise virus passaging [28] or continuous virus cultivation [24], will have an impact on the results and make these analyses more complex.

These differences of the experimental setup could additionally impact the analyzed DelVG populations. For instance, batch effects can influence the results of omics data analysis, as shown in recent papers [61, 62]. But with very distinct and relatively weakly overlapping sets of DelVGs, the detection of batch effects is challenging. Despite this, we have looked into the detection of possible batch effects of the DelVG counts by Uniform Manifold Approximation and Projection and sequence clustering (Supplementary Fig. S10). The approaches indicate only strain and segment specificity, but no clear batch effects. However, this observation is due to the unique combination of virus strain, assay system, organism, and experimental setup per dataset. Therefore, our analyses do not rule out the presence of possible batch effects, and should rather motivate the development of batch effect detection and correction methods for DelVG research in upcoming work.

In summary, we assembled a more comprehensive comparison across different IAV and IBV DelVG datasets originating from different laboratories and publications, by applying the same methods to calculate DelVG features, such as sequence length, and nucleotide enrichment. Our findings indicate a difference in the lengths of 3′- and 5′-end sequences of IAV and IBV strains. Furthermore, our analysis reveals that DelVGs from *in vivo* experiments tend to be longer than those from *in vitro* experiments. The distribution of direct repeats is consistent for all datasets, except for the dataset including A/California/07/2009. Further research is required to clarify if this difference is dataset- or strain-specific. Finally, we used the A/Puerto Rico/8/1934 datasets of our meta-analysis to identify 11 DelVG candidates of potential relevance for medical application.

## Supplementary Material

lqaf031_Supplemental_File

## Data Availability

The data underlying this article are available in the article and in its online supplementary material. Original code for data analysis including the underlying datasets is available on Zenodo (https://doi.org/10.5281/zenodo.14872995) or GitHub (https://github.com/viraidip/DIP_meta-study). An R shiny application to analyze additional datasets is available on Zenodo (https://doi.org/10.5281/zenodo.12157628) or GitHub (https://github.com/viraidip/DIP-DSA).
