# Supplementary material for: Meta-analysis of genomic characteristics for antiviral influenza defective interfering particle prioritization
Source: NAR Genom Bioinform. 2025 Apr 4;7(2):lqaf031. doi: 10.1093/nargab/lqaf031 (PMC11970370; doi:10.1093/nargab/lqaf031)
Supplement: lqaf031_Supplemental_File [file lqaf031_supplemental_file.pdf]

Supplementary data for the article:

Meta-analysis of genomic characteristics for antiviral influenza defective interfering particle prioritization

AUTHORS

Jens J. G. Lohmann[1], Mia Le[1,2], Fadi G. Alnaji[3], Olga Zolotareva[1], Jan Baumbach[1,4,†], and Tanja Laske[1,5,*,†]

[1] Institute for Computational Systems Biology, University of Hamburg, 22607 Hamburg, Germany
[2] Department of Virology, Bernhard Nocht Institute for Tropical Medicine, 20359 Hamburg, Germany
[3] A*STAR Infectious Diseases Labs (A*STAR ID Labs), Agency for Science, Technology and Research (A*STAR), 8A Biomedical Grove, Immunos #05-13, Singapore, 138648, Singapore
[4] Computational Biomedicine Lab, Department of Mathematics and Computer Science, University of Southern Denmark, 5230 Odense, Denmark
[5] Leibniz Institute of Virology, 20251 Hamburg, Germany

* To whom correspondence should be addressed. Email: tanja.laske@uni-hamburg.de
† The last two authors should be regarded as Joint Last Authors.

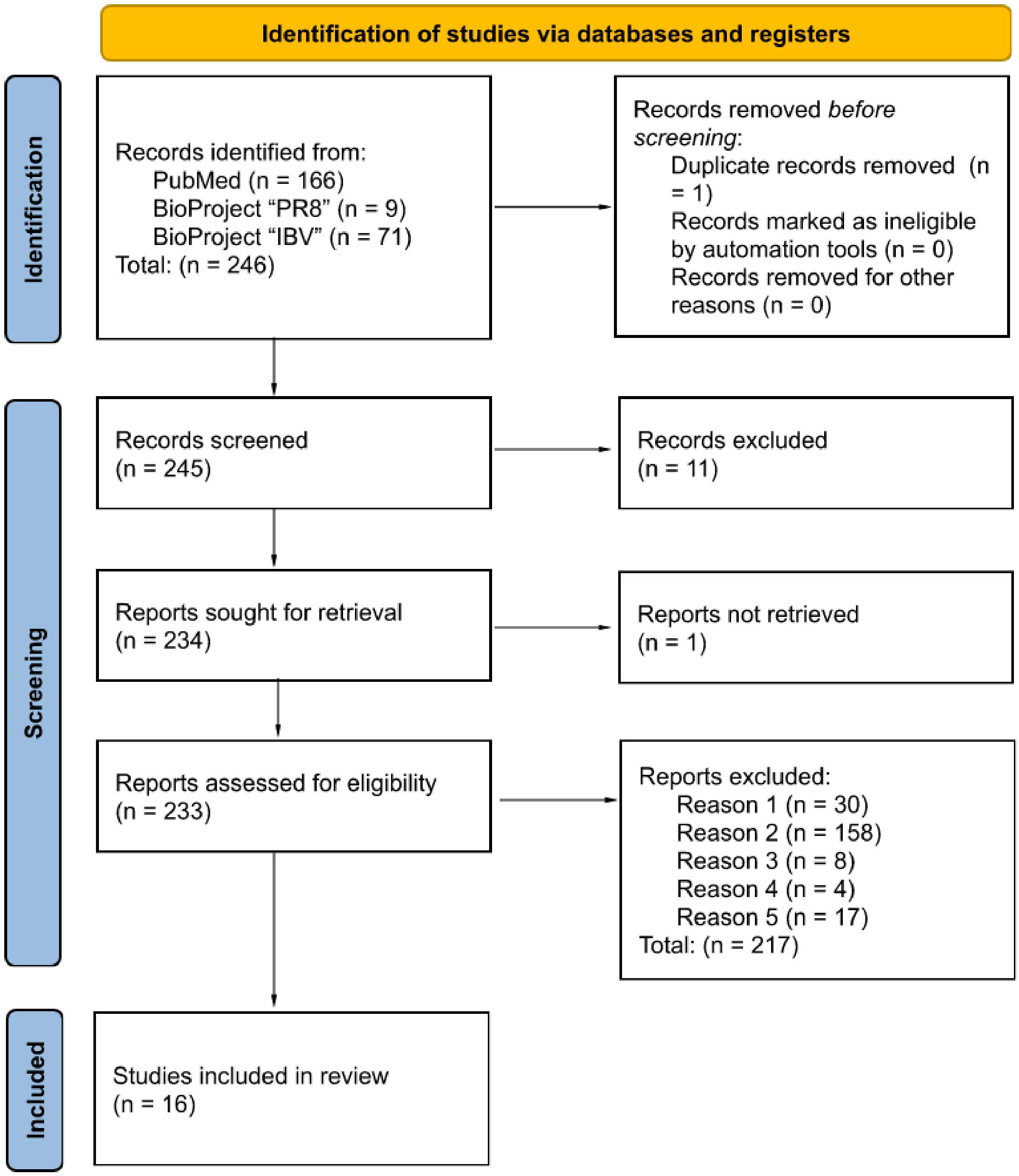

Supplementary Figure 1: PRISMA (1) flow chart for the queries. Three different searches were performed, and the hits were filtered. The exclusion criteria were the following: R1: the entry includes no DelVG/virus population, R2: no RNA-seq data available, R3: the virus coverage of the dataset is < 2 %, R4: the number of reads in the datasets is infeasible (below 100 or above 50 million), R5: less than 50 DelVGs were found with the pipeline (2).

Supplementary Table 1: List of sources and metadata of the considered NGS datasets. Data for Penn2022 (3) was retrieved from EBI (www.ebi.ac.uk). For Boussier2020 (4) raw data was retrieved directly from the authors. The last column corresponds to the number of individual entries that we used from the BioProject or EBI project to build the overall dataset.

Dataset name	BioProject or EBI ID	Considered strain	Subtype	Assay system	Number of individual datasets
Alnaji2021 (5)	PRJNA725907	A/Puerto Rico/8/1934/Mount Sinai	H1N1	*in vitro* (MDCK-SIAT1)	10
Pelz2021 (6)	PRJNA743179	A/Puerto Rico/8/1934/Mount Sinai	H1N1	*in vitro* (MDCK)	23
Wang2023 (7)	PRJNA777796	A/Puerto Rico/8/1934/Mount Sinai	H1N1	*in vivo* mouse	28
Wang2020 (8)	PRJNA486793	A/Puerto Rico/8/1934/Mount Sinai	H1N1	*in vitro* (A549, HBEpC)	12
Zhuravlev2020 (9)	PRJEB40202	A/Puerto Rico/8/1934/Mount Sinai	H1N1	*in vitro* (A549, HEK293FT, MRC5, WI38)	8
Kupke2020 (10)	PRJNA590388	A/Puerto Rico/8/1934/Mount Sinai	H1N1	*in vitro* (MDCK)	98
VdHoecke2015 (11)	PRJNA272588	A/Puerto Rico/8/1934/Mount Sinai	H1N1	*in vitro* (MDCK)	2
Alnaji2019_Cal07 (2)	PRJNA527853	A/California/07/2009	H1N1	*in vitro* (MDCK)	5
Alnaji2019_NC (2)	PRJNA527853	A/New Caledonia/20-JY2/1999	H1N1	*in vitro* (MDCK)	4
Mendes2021 (12)	PRJNA760790	A/WSN/1933	H1N1	*in vitro* (A549)	8
Boussier2020 (4)	-	A/WSN/1933	H1N1	*in vitro* (MDCK)	23
Alnaji2019_Perth (2)	PRJNA527853	A/Perth/16/2009	H3N2	*in vitro* (MDCK)	4
Berry2021_A (13)	PRJNA747705	A/Connecticut/Flu122/2013	H3N2	*in vivo* human	8
Penn2022 (3)	PRJEB56225	A/turkey/Turkey/1/2005	H5N1	*in vivo* mouse	16
Lui2019 (14)	PRJNA421629	A/Anhui/1/2013	H7N9	*in vivo* mouse	2
Alnaji2019_BLEE (2)	PRJNA527853	B/Lee/1940	IBV	*in vitro* (MDCK)	4
Berry2021_B (13)	PRJNA747705	B/Victoria/504/2000	IBV	*in vivo* human	22
Valesano2020_Vic (15)	PRJNA561158	B/Victoria/504/2000	IBV	*in vivo* human	19
Sheng2018 (16)	PRJNA314541	B/Brisbane/60/2008	IBV	*in vitro* (A549)	7
Berry2021_B_Yam (13)	PRJNA747705	B/Yamagata/16/1988	IBV	*in vivo* human	6
Southgate2019 (17)	PRJEB33950	B/Yamagata/16/1988	IBV	*in vivo* human	58
Valesano2020_Yam (15)	PRJNA561158	B/Yamagata/16/1988	IBV	*in vivo* human	24

Supplementary Table 2: Accession numbers considered for the different strains.

Strain	PB2	PB1	PA	HA	NP	NA	M	NS
A/Puerto Rico/8/34/Mount Sinai	AF389115.1	AF389116.1	AF389117.1	AF389118.1	AF389119.1	AF389120.1	AF389121.1	AF389122.1
A/California/07/2009	CY121687.1	CY121686.1	CY121685.1	CY121680.1	CY121683.1	CY121682.1	CY121681.1	CY121684.1
A/New Caledonia/20-JY2/1999	CY147325.1	CY147324.1	CY147323.1	CY147318.1	CY147321.1	CY147320.1	CY147319.1	CY147322.1
A/Perth/16/2009	KJ609203.1	KJ609204.1	KJ609205.1	KJ609206.1	KJ609207.1	KJ609208.1	KJ609209.1	KJ609210.1
A/Connecticut/Flu122/2013	KM654658.1	KM654706.1	KM654754.1	KM654822.1	KM654847.1	KM654920.1	KM654969.1	KM654612.1
A/turkey/Turkey/1/2005	EF619975.1	EF619976.1	EF619979.1	AF389118.1	EF619977.1	EF619973.1	EF619978.1	EF619974.1
A/Anhui/1/2013	EPI439504	EPI439508	EPI439503	EPI439507	EPI439505	EPI439509	EPI439506	EPI439510
B/Lee/1940	CY115118.1	CY115117.1	CY115116.1	CY115111.1	CY115114.1	CY115113.1	CY115112.1	CY115115.1
B/Victoria/504/2000	CY018660.1	CY018659.1	CY018658.1	CY018653.1	CY018656.1	CY018655.1	CY018654.1	CY018657.1
B/Yamagata/16/1988	OQ034430.1	OQ034429.1	OQ034431.1	OQ034432.1	OQ034433.1	OQ034434.1	OQ034435.1	OQ034436.1
A/WSN/1933	LC333182.1	LC333183.1	LC333184.1	LC333185.1	LC333186.1	LC333187.1	LC333188.1	LC333189.1

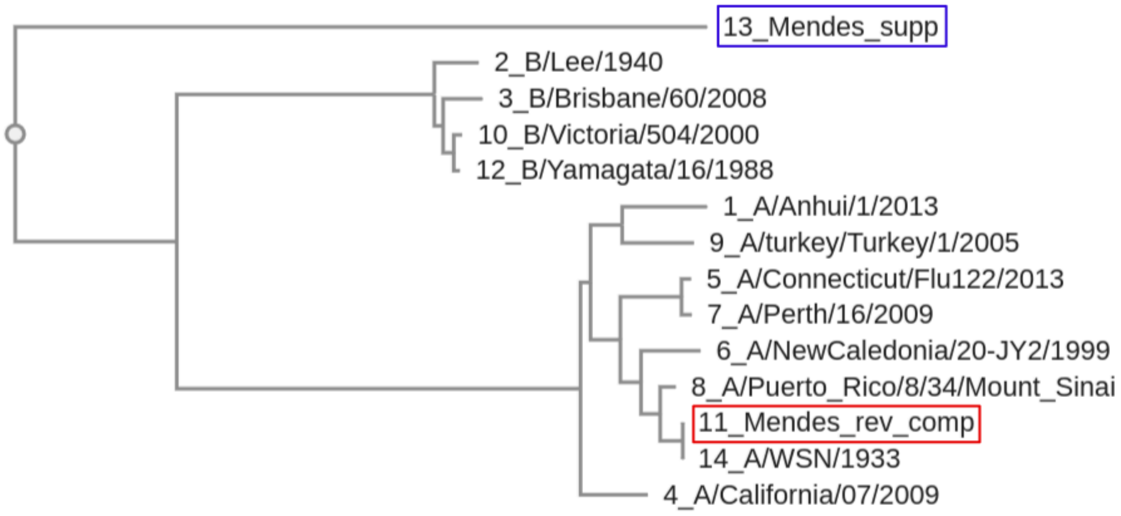

Supplementary Figure 2: Verification of A/WSN/1933 reference sequences by a multiple sequence alignment (MSA). The MSA was performed using MAFFT (mafft.cbrc.jp). The sequences provided in the supplement by Mendes et al. (12) (*13_Mendes_supp*, blue) are not located next to the influenza A virus sequences. The reverse complement of these sequences (*11_Mendes_rev_comp*, red) is located directly next to the A/WSN/1933 reference sequence of PB2 (*14_A/WSN/1993*, LC333182.1). Therefore, the reverse complements of the sequences were used in the analysis.

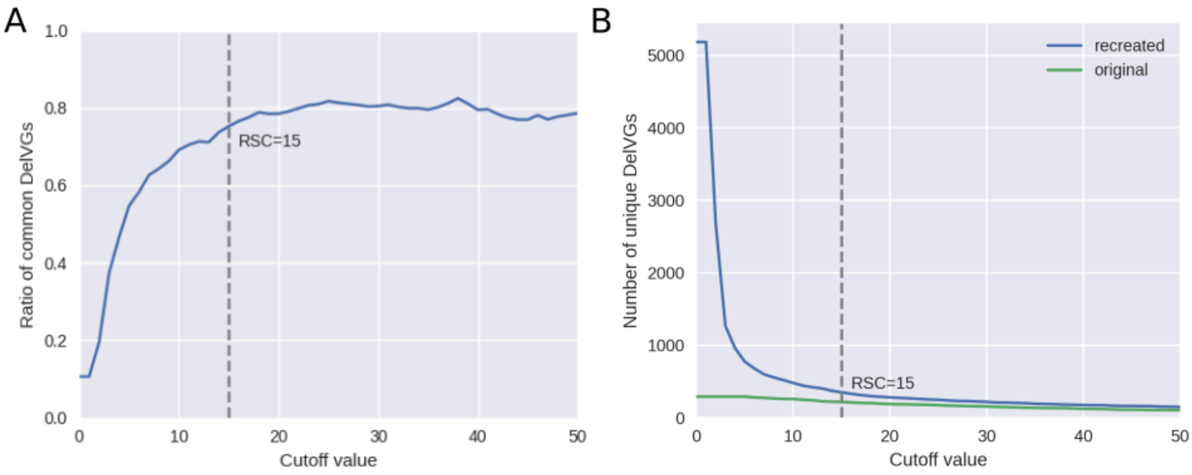

Supplementary Figure 3: Read support cutoff (RSC) estimation for Alnaji2019_BLEE (2). (A) The ratio of common DelVGs between the original and the processed dataset is given for an increasing cutoff. The RSC with a Dice coefficient higher than 0.75 was estimated (gray line). (B) Number of DelVGs for the original dataset (green) and the recreated dataset (blue). The recreated dataset contains more DelVGs when no cutoff is applied. The estimated RSC is given in a gray line.

Supplementary Table 3: Estimated read support cutoff (RSC) for datasets that were provided with the original publication. The estimation was done as described with Figure S3.

**Publication**	**Dataset**	**Read support cutoff (RSC)**
Pelz et al. (2021) (6)	Pelz2021	11
Alnaji et al. (2021) (5)	Alnaji2021	6
Alnaji et al. (2019) (2)	Alnaji2019_Cal07	13
Alnaji et al. (2019) (2)	Alnaji2019_NC	12
Alnaji et al. (2019) (2)	Alnaji2019_Perth	9
Alnaji et al. (2019) (2)	Alnaji2019_BLEE	15

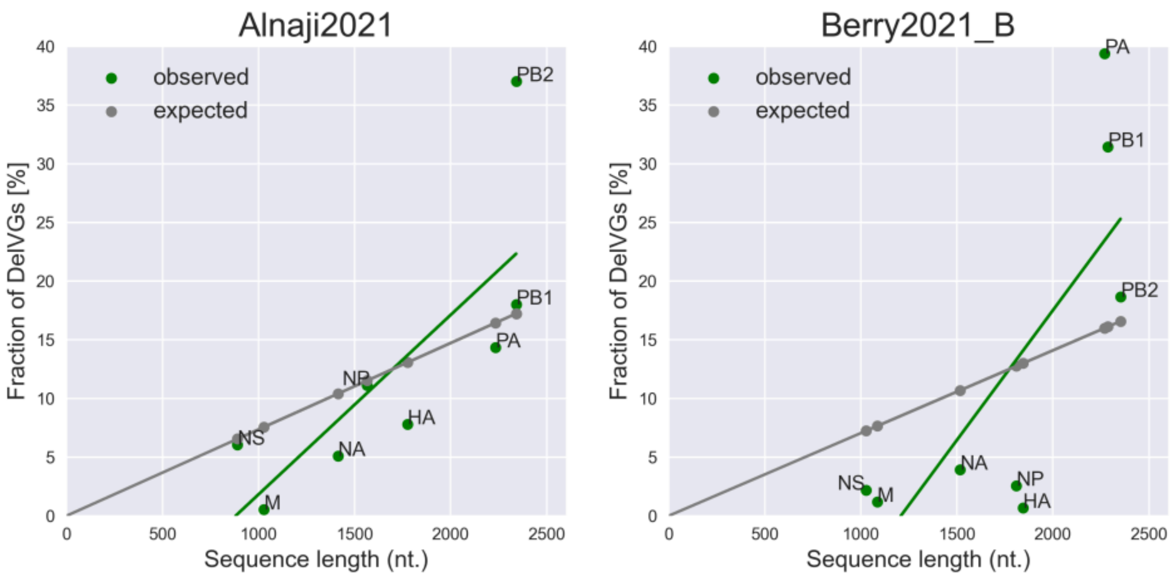

Supplementary Figure 4: Segment distribution for Alnaji2021 (5) and Berry2021_B (13) compared to distribution based on full segment length. The observed percentages of the DelVGs per segment (green) was compared to the expected (gray). For this, we assumed that the length of the wild type sequence is proportional to the number of formed DelVGs. Hence, the longer a segment is, the more DelVGs are formed. The two examples show that the observed DelVG distribution does not follow this expected distribution. The results for the chi-squared test are X^2=461.9, p<0.001, Cramer's V=0.32 for Alnaji2021 and X^2=137.7, p<0.001, Cramer's V=0.39 for Berry2021_B.

Supplementary Table 4: Overview of the number of all mapped DelVG sequences split into the two categories of host system and influenza virus type.

	in vitro	*mouse*	*human*	overall
**pooled data**				
**IAV**	11517	6492	1080	19089
**IBV**	541	0	3735	4276
**overall**	12058	6492	4815	23365
**PB2**				
**IAV**	2621	1209	102	3932
**IBV**	101	0	1186	1287
**overall**	2722	1209	1288	5219
**PB1**				
**IAV**	4289	2518	161	6968
**IBV**	83	0	1512	1595
**overall**	4372	2518	1673	8563
**PA**				
**IAV**	3416	2560	689	6665
**IBV**	77	0	830	907
**overall**	3493	2560	1519	7572

Supplementary Table 5: Results for Wilcoxon-Mann-Whitney U test.

	**in vitro - mouse**	**in vitro - human**	**mouse - human**
DelVG length			
**PB2** (n=5219)	U=1250954.0 p=3.0 * 10^-33	U=939684.5 p=9.1 * 10^-125	U=584813.0 p=5.1 * 10^-27
**PB1** (n=8563)	U=4358330.5 p=4.2 * 10^-47	U=2215338.5 p=4.2 * 10^-124	U=1541022 p=3.8*10^-49
**PA** (n=7572)	U=3624901 p=2.1 * 10^-36	U=1886535.5 p=1.4 * 10^-59	U=1754845 p=1.9 * 10^-7

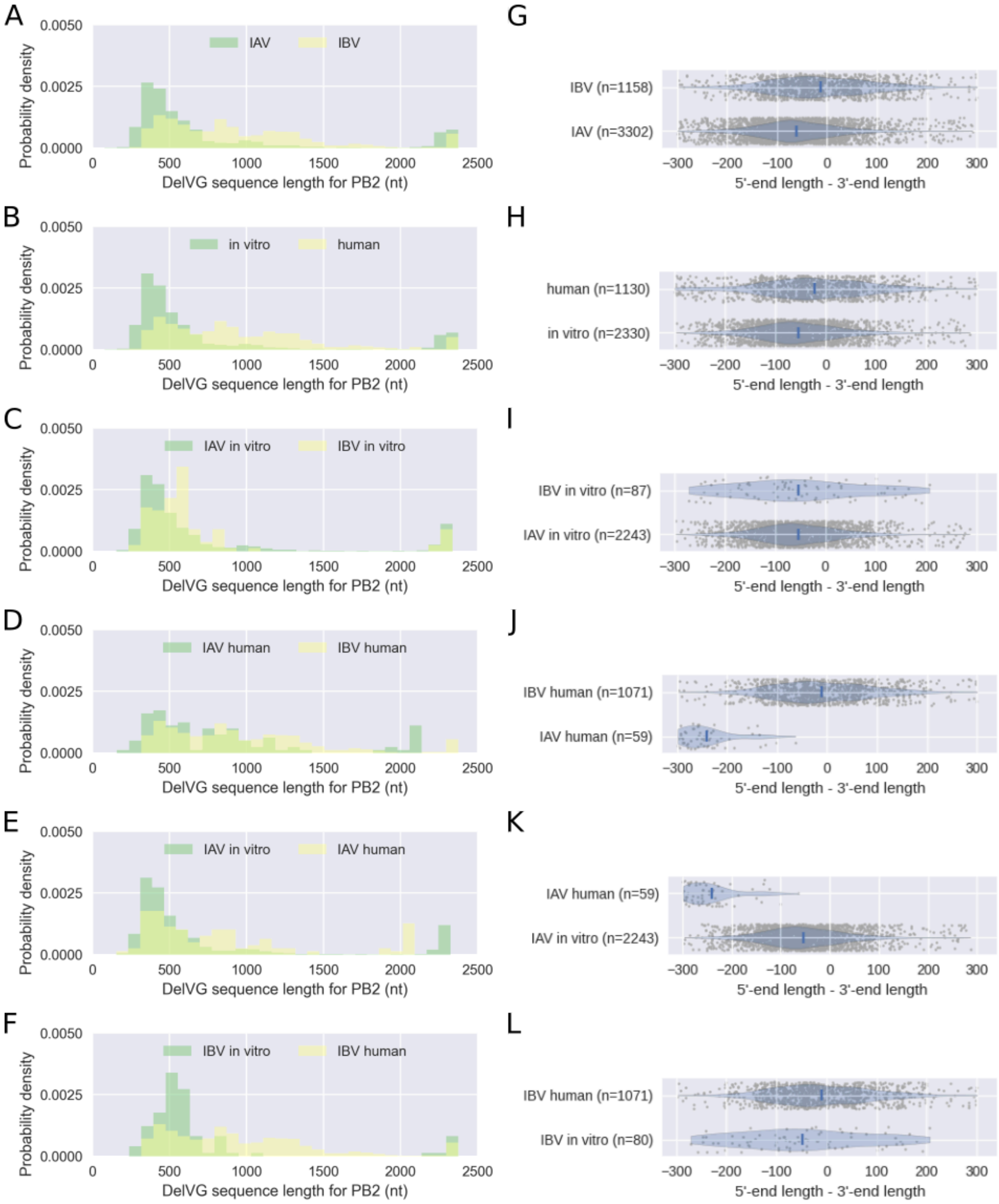

Supplementary Figure 5: Comparison of the subgroups of influenza virus type and host system on DelVG differences (PB2). The distributions of DelVG sequence lengths (left, A-F) and of the differences in 3'- and 5'-end lengths (right, G-L). Comparison with respect to influenza virus type (A/G) or host systems (B/H). Comparison of influenza virus type, considering either *in vitro* (C/I) or human (D/J) datasets. Comparison of host systems, considering either IAV (E/K) or IBV (F/L) datasets.

Supplementary Table 6: Results for Scheirer-Ray-Hare tests. Significant p-values were highlighted after adjustment by Bonferroni correction (α=0.05/4=0.0125) for DelVG length and differences of 3'-5' ends.

	**Influenza type**	**Host system**	**Interaction**
DelVG length			
**Pooled** (n=23365)	H=1.93 p=0.1651	H=6.91 p=0.0086	H=7.16 p=0.0074
**PB2** (n=5219)	H=3.55 p=0.0596	H=7.47 p=0.0063	H=4.98 p=0.0257
**PB1** (n=8563)	H=0.8 p=0.3722	H=4.88 p=0.0272	H=10.33 p=0.0013
**PA** (n=7572)	H=3.18 p=0.0743	H=9.3 p=0.0023	H=3.52 p=0.0608
3'-5' length			
**Pooled** (n=23365)	H=9.45 p=0.0021	H=5.35 p=0.0207	H=1.2 p=0.273
**PB2** (n=5219)	H=4.76 p=0.0292	H=5.35 p=0.0207	H=5.89 p=0.0152
**PB1** (n=8563)	H=7.71 p=0.0055	H=4.42 p=0.0355	H=3.87 p=0.0491
**PA** (n=7572)	H=6.65 p=0.0099	H=4.42 p=0.0355	H=4.93 p=0.0264

Supplementary Table 7: Occurrence of long DelVG candidates (> 85 % of sequence retained) in all influenza virus genome segments. Datasets are listed in descending order according to their content of long DelVGs.

Dataset	Number of long DelVG candidates	Percentage of long DelVG candidates
Lui2019 (27)	14	26.4
Boussier2020 (28)	86	25.5
Pelz2021 (24)	1438	23.0
Berry2021_A (33)	215	19.9
Wang2020 (39)	40	18.7
Wang2023 (37)	1172	18.6
Zhuravlev2020 (31)	7	14.3
Alnaji2019_NC (29)	40	11.9
Mendes2021 (38)	113	9.2
Penn2022 (40)	12	8.8
Valesano2020_Vic (34)	5	6.6
Berry2021_B (33)	14	6.0
Berry2021_B_Yam (33)	32	4.2
Southgate2019 (35)	84	4.0
Alnaji2021 (25)	53	3.9
Valesano2020_Yam (34)	20	3.7
Sheng2018 (36)	2	3.6
Alnaji2019_BLEE (29)	17	3.5
Alnaji2019_Perth (29)	21	2.1
Alnaji2019_Cal07 (29)	5	0.7

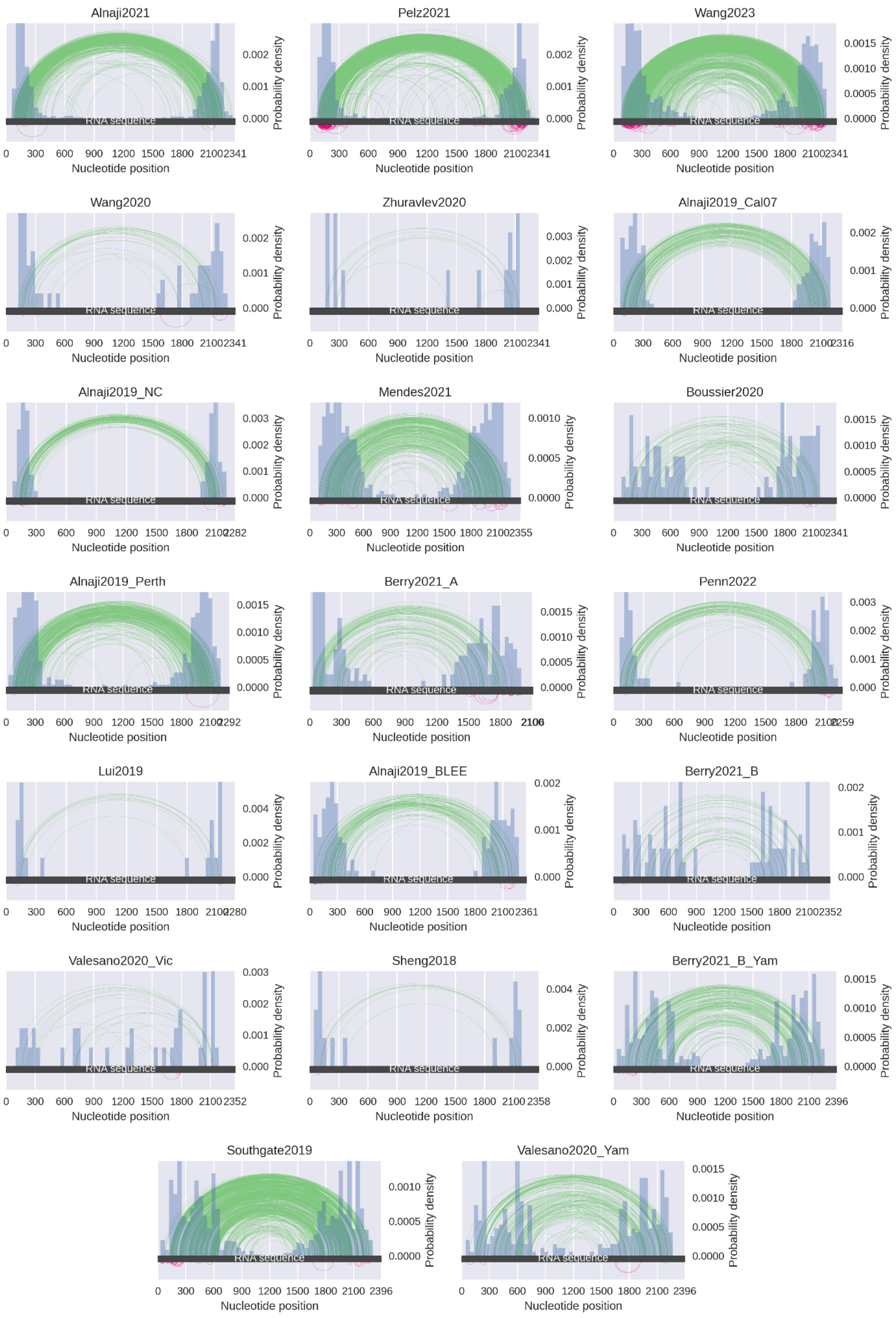
Alnaji2021
Pelz2021
Wang2023
Wang2020
Zhuravlev2020
Alnaji2019_Cal07
Alnaji2019_NC
Mendes2021
Boussier2020
Alnaji2019_Perth
Berry2021_A
Penn2022
Lui2019
Alnaji2019_BLEE
Berry2021_B
Valesano2020_Vic
Sheng2018
Berry2021_B_Yam
Southgate2019
Valesano2020_Yam
RNA sequence
Nucleotide position
Probability density

Supplementary Figure 6: Mapping of start and end position of deletion site for PB2 segment. Distribution of the deletion site positions is given in a histogram in blue. Long DelVGs with a sequence length of at least 85 % of the full sequence are marked in pink (below the RNA sequence). All other DelVGs are marked in green (above the RNA sequence).

Supplementary Table 8: Overview of the 3'- 5'- differences for the different analyzed data splits.

**Comparison**	**3'-5'-diff** (factor 1)	**3'-5'-diff** (factor 2)	**Total difference**	**Label in Figure S6**
Factor 1: IAV<br>Factor 2: IBV	-66 nt.	-12 nt.	54 nt.	G
Factor 1: *in vitro*<br>Factor 2: human	-62 nt.	-24 nt.	38 nt.	H
Factor 1: IAV *in vitro*<br>Factor 2: IBV *in vitro*	-64 nt.	-38 nt.	26 nt.	I
Factor 1: IAV human<br>Factor 2: IBV human	-93 nt.	-9 nt.	84 nt.	J
Factor 1: IAV *in vitro*<br>Factor 2: IBV human	-64 nt.	-93 nt.	-29 nt.	K
Factor 1: IBV *in vitro*<br>Factor 2: IAV human	-38 nt.	-9 nt.	29 nt.	L

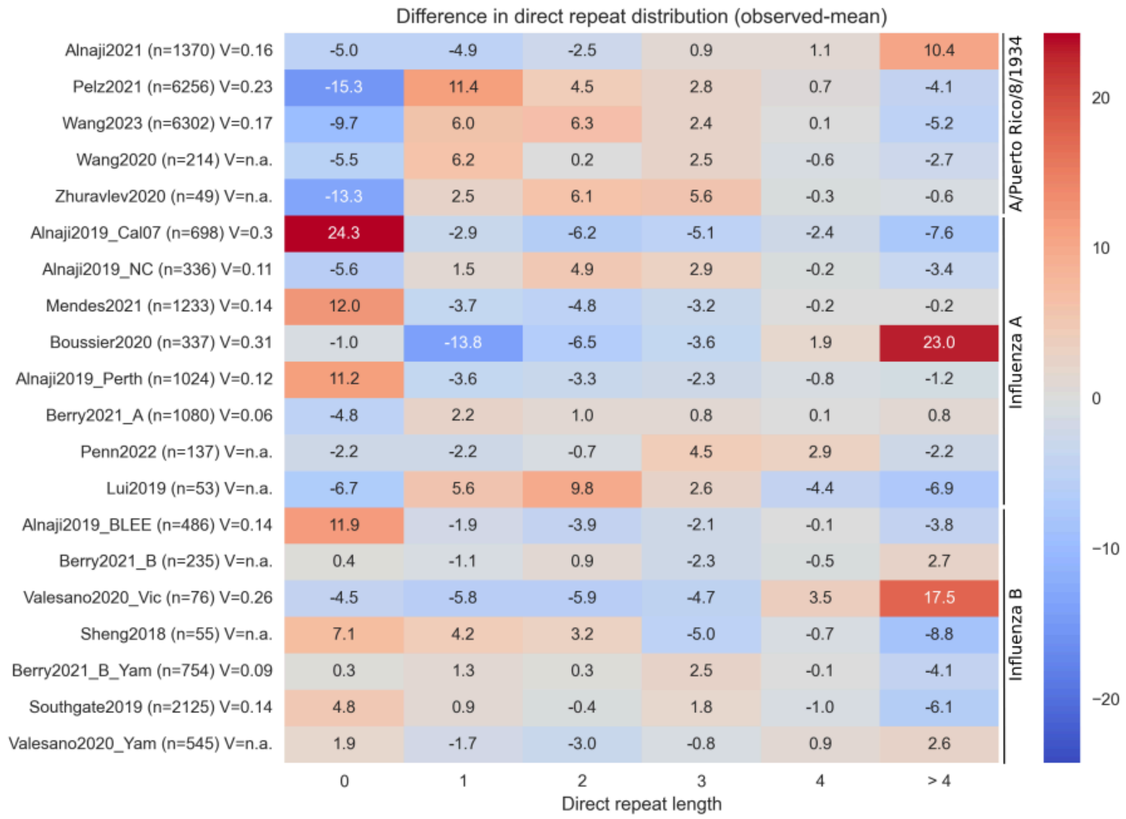

	0	1	2	3	4	> 4
Alnaji2021 (n=1370) V=0.16	-5.0	-4.9	-2.5	0.9	1.1	10.4
Pelz2021 (n=6256) V=0.23	-15.3	11.4	4.5	2.8	0.7	-4.1
Wang2023 (n=6302) V=0.17	-9.7	6.0	6.3	2.4	0.1	-5.2
Wang2020 (n=214) V=n.a.	-5.5	6.2	0.2	2.5	-0.6	-2.7
Zhuravlev2020 (n=49) V=n.a.	-13.3	2.5	6.1	5.6	-0.3	-0.6
Alnaji2019_Cal07 (n=698) V=0.3	24.3	-2.9	-6.2	-5.1	-2.4	-7.6
Alnaji2019_NC (n=336) V=0.11	-5.6	1.5	4.9	2.9	-0.2	-3.4
Mendes2021 (n=1233) V=0.14	12.0	-3.7	-4.8	-3.2	-0.2	-0.2
Boussier2020 (n=337) V=0.31	-1.0	-13.8	-6.5	-3.6	1.9	23.0
Alnaji2019_Perth (n=1024) V=0.12	11.2	-3.6	-3.3	-2.3	-0.8	-1.2
Berry2021_A (n=1080) V=0.06	-4.8	2.2	1.0	0.8	0.1	0.8
Penn2022 (n=137) V=n.a.	-2.2	-2.2	-0.7	4.5	2.9	-2.2
Lui2019 (n=53) V=n.a.	-6.7	5.6	9.8	2.6	-4.4	-6.9
Alnaji2019_BLEE (n=486) V=0.14	11.9	-1.9	-3.9	-2.1	-0.1	-3.8
Berry2021_B (n=235) V=n.a.	0.4	-1.1	0.9	-2.3	-0.5	2.7
Valesano2020_Vic (n=76) V=0.26	-4.5	-5.8	-5.9	-4.7	3.5	17.5
Sheng2018 (n=55) V=n.a.	7.1	4.2	3.2	-5.0	-0.7	-8.8
Berry2021_B_Yam (n=754) V=0.09	0.3	1.3	0.3	2.5	-0.1	-4.1
Southgate2019 (n=2125) V=0.14	4.8	0.9	-0.4	1.8	-1.0	-6.1
Valesano2020_Yam (n=545) V=n.a.	1.9	-1.7	-3.0	-0.8	0.9	2.6

Supplementary Figure 7: Comparison of the direct repeat lengths against dataset mean. The percentages of the calculated direct repeat lengths were compared to the mean over all datasets. By this the differences between different strains or datasets can be elaborated in more detail. Chi-squared test and Cramer's V was applied for statistical analysis.

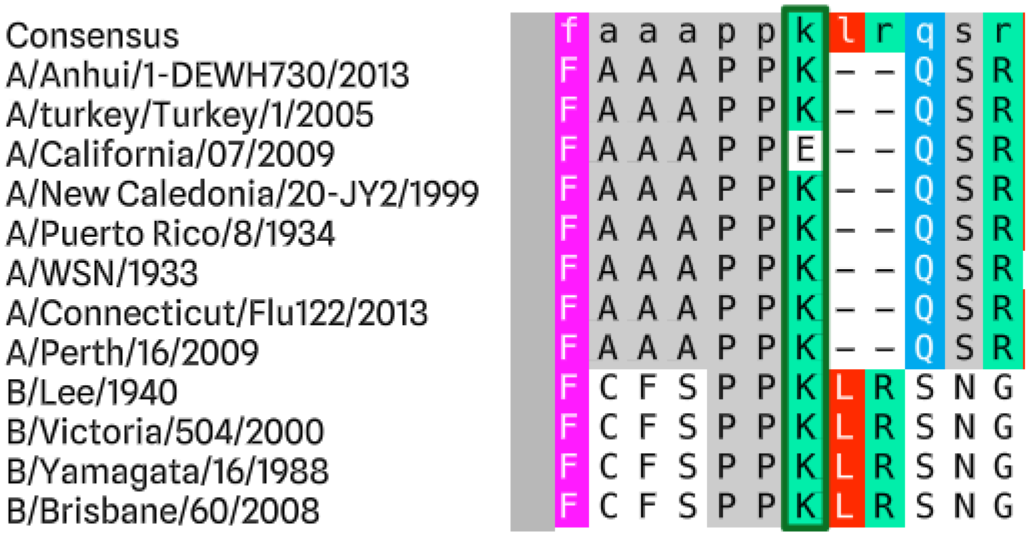

Supplementary Figure 8: Multiple sequence alignment of the PB2 protein sequences for all considered strains. Part of the multiple sequence alignment (MSA) performed for the PB2 protein sequences of the considered strains using MAFFT (mafft.cbrc.jp). The green box indicates mutation K627E on the A/California/07/2009 strain.

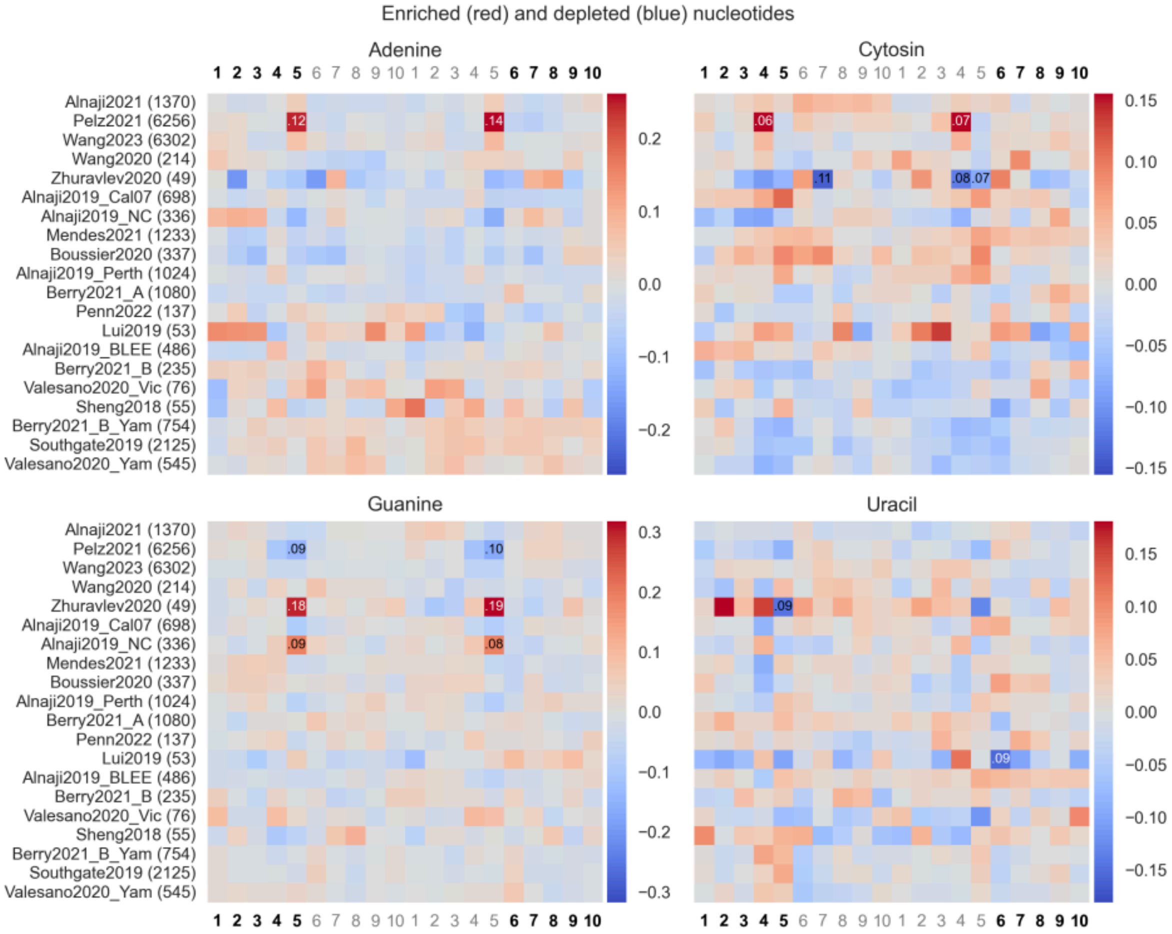

Supplementary Figure 9: Comparison of enriched nucleotides compared to the dataset mean. The difference of the calculated frequencies per dataset was compared to the mean over all datasets. Statistical significance between the frequencies per dataset and mean frequencies was estimated by Kruskal-Wallis test and for positions with significant p-values ($p<0.05$) the effect size η^2 was reported for positions with an at least medium effect ($\eta^2 \geq 0.06$).

Supplementary Table 9: Comparison of the most frequent nucleotide pairs at position 4 and 5 of the start and end of the deletion site. As reference, synthetic data was generated (Section "Random sampling approach to generate synthetic datasets") and the most frequent nucleotide pair was estimated.

	Observed data				Synthetic data			
**Dataset**	**Start**	**Occurrence [%]**	**End**	**Occurrence [%]**	**Start**	**Occurrence [%]**	**End**	**Occurrence [%]**
Alnaji2021 (5)	UA	16.1	UA	16.4	AA	15.5	AA	12.0
Pelz2021 (6)	CA	30.6	CA	27.3	AA	15.7	AA	13.0
Wang2023 (7)	CA	18.8	CA	16.9	AA	14.7	AA	13.9
Wang2020 (8)	UA	14.0	UA	14.0	AA	15.1	AA	12.6
Zhuravlev2020 (9)	UG	34.7	UG	22.4	AA	15.7	AA	11.6
Alnaji2019_Cal07 (2)	AA	13.3	AA	14.6	AA	13.7	AA	12.6
Alnaji2019_NC (2)	UG	23.5	UG	19.0	AA	16.4	AA	12.5
Mendes2021 (12)	GA	11.4	GA	11.7	AA	16.2	AA	13.9
Boussier2020 (4)	AA	11.0	GA	11.0	AA	14.6	AA	12.9
Alnaji2019_Perth (2)	AA	13.5	AA	12.7	AA	15.2	AA	13.5
Berry2021_A (13)	UA	16.3	UA	14.9	AA	14.1	AA	13.9
Penn2022 (3)	GA	17.5	GA	17.5	AA	12.8	AA	13.0
Lui2019 (14)	UA	17.0	UG	18.9	AA	12.0	AA	11.0
Alnaji2019_BLEE (2)	AA	16.0	UA	14.0	AA	17.6	AA	14.4
Berry2021_B (13)	UA	14.9	UA	14.9	AA	16.1	AA	15.0
Valesano2020_Vic (15)	UA	15.8	GA	18.4	AA	16.4	AA	14.3
Sheng2018 (16)	UA	18.2	AA	21.8	AA	19.5	AA	15.4
Berry2021_B_Yam (13)	UA	21.1	AA	15.0	AA	18.5	AA	16.0
Southgate2019 (17)	UA	15.8	AA	15.6	AA	19.1	AA	15.0
Valesano2020_Yam (15)	UA	16.3	UA	14.1	AA	16.5	AA	14.9

Supplementary Table 10: Identified top DelVG candidates for the polymerase segments.

DelVG	Found in # dataset	Label	Score sum	Score mean	DelVG length	Frame shift	Direct repeat	Nucleotide pair start	Nucleotide pair end	Δ 5’-3’ end	*in vivo*
PA_138_2063	5	loss	481.9	96.4	308	+1	1	UA	GA	-32	yes
PA_139_169	5	gain	485.0	97.0	2204	-1	3	AU	AU	-1925	yes
PA_167_1990	6	gain	573.2	95.5	411	+1	2	UA	UA	-76	yes
PB1_54_98	5	loss	473.1	94.6	2298	+1	2	UA	UA	-2189	yes
PB1_113_2165	5	gain	483.5	96.7	290	-1	2	UA	UA	-63	yes
PB1_200_2072	5	gain	482.8	96.6	470	-1	2	AC	AC	-69	yes
PB2_109_2152	5	gain	470.1	94.0	299	-1	2	UA	UA	-80	yes
PB2_163_2139	5	*de novo* gain	462.8	92.6	366	+1	2	UA	UA	-39	yes
PB2_163_2152	6	*de novo* gain	567.6	94.6	353	-1	2	UA	UA	-26	yes
PB2_177_2141	5	*de novo* loss	462.0	92.4	378	+1	3	UG	UG	-23	yes
PB2_191_2048	5	*de novo* gain	467.7	93.5	485	-1	1	UA	CA	-102	yes

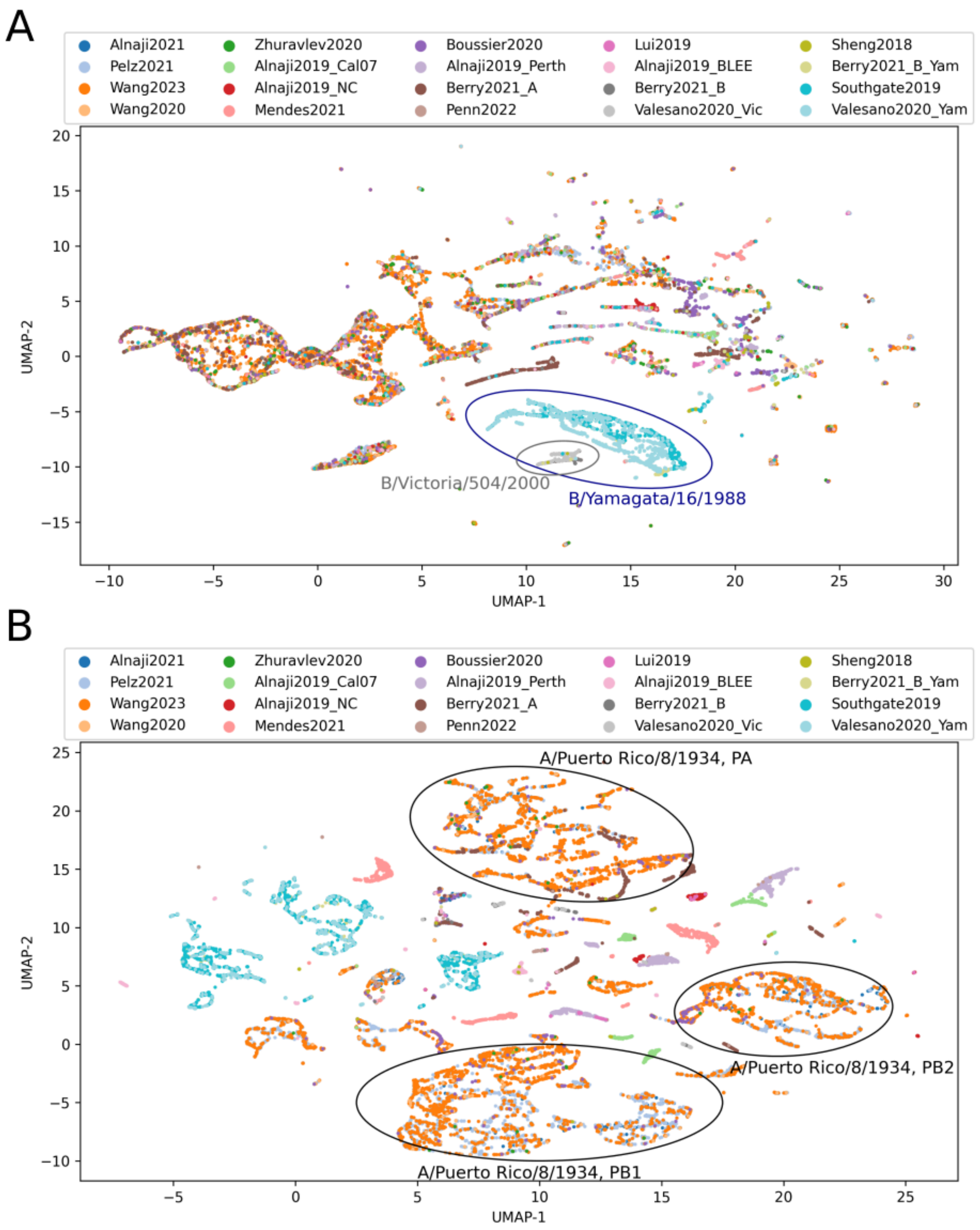

Supplementary Figure 10: Assessment of possible batch effects. (A) Uniform Manifold Approximation and Projection (UMAP) of the raw DelVG NGS counts across the twenty selected datasets. To reduce the sparsity only DelVGs were considered that occur in at least two of the datasets. The two clustering IBV strains B/Yamagata/16/1988 (blue) and B/Victoria/504/2000 (grey) are highlighted. (B) UMAP of the one-hot encoded DelVG sequences. The polymerase segments for the A/Puerto Rico/8/1934 strain (black) are highlighted.

REFERENCES FOR SUPPLEMENTARY MATERIAL

1. Page,M.J., McKenzie,J.E., Bossuyt,P.M., Boutron,I., Hoffmann,T.C., Mulrow,C.D., Shamseer,L., Tetzlaff,J.M., Akl,E.A., Brennan,S.E., *et al.* (2021) The PRISMA 2020 statement: an updated guideline for reporting systematic reviews. *BMJ*, **372**, n71.
2. Alnaji,F.G., Holmes,J.R., Rendon,G., Vera,J.C., Fields,C.J., Martin,B.E. and Brooke,C.B. (2019) Sequencing Framework for the Sensitive Detection and Precise Mapping of Defective Interfering Particle-Associated Deletions across Influenza A and B Viruses. *J. Virol.*, **93**.
3. Penn,R., Tregoning,J.S., Flight,K.E., Baillon,L., Frise,R., Goldhill,D.H., Johansson,C. and Barclay,W.S. (2022) Levels of Influenza A Virus Defective Viral Genomes Determine Pathogenesis in the BALB/c Mouse Model. *J. Virol.*, **96**, e0117822.
4. Boussier,J., Munier,S., Achouri,E., Meyer,B., Crescenzo-Chaigne,B., Behillil,S., Enouf,V., Vignuzzi,M., van der Werf,S. and Naffakh,N. (2020) RNA-seq accuracy and reproducibility for the mapping and quantification of influenza defective viral genomes. *RNA N. Y. N*, **26**, 1905–1918.
5. Alnaji,F.G., Reiser,W.K., Rivera-Cardona,J., Te Velthuis,A.J.W. and Brooke,C.B. (2021) Influenza A Virus Defective Viral Genomes Are Inefficiently Packaged into Virions Relative to Wild-Type Genomic RNAs. *mBio*, **12**, e0295921.
6. Pelz,L., Rüdiger,D., Dogra,T., Alnaji,F.G., Genzel,Y., Brooke,C.B., Kupke,S.Y. and Reichl,U. (2021) Semi-continuous Propagation of Influenza A Virus and Its Defective Interfering Particles: Analyzing the Dynamic Competition To Select Candidates for Antiviral Therapy. *J. Virol.*, **95**, e0117421.
7. Wang,C., Honce,R., Salvatore,M., Chow,D., Randazzo,D., Yang,J., Twells,N.M., Mahal,L.K., Schultz-Cherry,S. and Ghedin,E. (2023) Influenza Defective Interfering Virus Promotes Multiciliated Cell Differentiation and Reduces the Inflammatory Response in Mice. *J. Virol.*, **97**, e0049323.
8. Wang,C., Forst,C.V., Chou,T., Geber,A., Wang,M., Hamou,W., Smith,M., Sebra,R., Zhang,B., Zhou,B., *et al.* (2020) Cell-to-Cell Variation in Defective Virus Expression and Effects on Host Responses during Influenza Virus Infection. *mBio*, **11**, 10.1128/mbio.02880-19.
9. Zhuravlev,E., Sergeeva,M., Malanin,S., Amirkhanov,R., Semenov,D., Grigoryeva,T., Komissarov,A. and Stepanov,G. (2020) RNA-Seq transcriptome data of human cells infected with influenza A/Puerto Rico/8/1934 (H1N1) virus. *Data Brief*, **33**, 106604.
10. Kupke,S.Y., Ly,L.-H., Börno,S.T., Ruff,A., Timmermann,B., Vingron,M., Haas,S. and Reichl,U. (2020) Single-Cell Analysis Uncovers a Vast Diversity in Intracellular Viral Defective Interfering RNA Content Affecting the Large Cell-to-Cell Heterogeneity in Influenza A Virus Replication. *Viruses*, **12**.
11. Van den Hoecke,S., Verhelst,J., Vuylsteke,M. and Saelens,X. (2015) Analysis of the genetic diversity of influenza A viruses using next-generation DNA sequencing. *BMC Genomics*, **16**, 79.
12. Mendes,M. and Russell,A.B. (2021) Library-based analysis reveals segment and length dependent characteristics of defective influenza genomes. *PLoS Pathog.*, **17**, e1010125.
13. Berry,I.M., Treangen,T., Fung,C., Tai,S., Pollett,S., Hong,F., Li,T., Pireku,P., Thomanna,A., German,J., *et al.* (2021) High confidence identification of intra-host single nucleotide variants for person-to-person influenza transmission tracking in congregate settings. *bioRxiv*, 10.1101/2021.07.01.450528.
14. Lui,W.-Y., Yuen,C.-K., Li,C., Wong,W.M., Lui,P.-Y., Lin,C.-H., Chan,K.-H., Zhao,H., Chen,H., To,K.K.W., *et al.* (2019) SMRT sequencing revealed the diversity and characteristics of defective interfering RNAs in influenza A (H7N9) virus infection. *Emerg. Microbes Infect.*, **8**, 662–674.
15. Valesano,A.L., Fitzsimmons,W.J., McCrone,J.T., Petrie,J.G., Monto,A.S., Martin,E.T. and Lauring,A.S. (2020) Influenza B Viruses Exhibit Lower Within-Host Diversity than Influenza A Viruses in Human Hosts. *J. Virol.*, **94**.
16. Sheng,Z., Liu,R., Yu,J., Ran,Z., Newkirk,S.J., An,W., Li,F. and Wang,D. (2018) Identification and

characterization of viral defective RNA genomes in influenza B virus. *J. Gen. Virol.*, **99**, 475–488.
17. Southgate,J.A., Bull,M.J., Brown,C.M., Watkins,J., Corden,S., Southgate,B., Moore,C. and Connor,T.R. (2020) Influenza classification from short reads with VAPOR facilitates robust mapping pipelines and zoonotic strain detection for routine surveillance applications. *Bioinformatics*, **36**, 1681–1688.
